# Prenatal Exposure to Fine Particulate Matter Components and Autism Risk in Childhood

**DOI:** 10.1001/jamanetworkopen.2025.38882

**Published:** 2025-10-23

**Authors:** Maxime Cloutier, Chengchun Yu, Robert Talarico, Steven Hawken, Hong Chen, Scott Weichenthal, Sabit Cakmak, Christopher Hebbern, Anna Gunz, Aaron van Donkelaar, Randall V. Martin, Jean-Nicolas Côté, Éric Lavigne

**Affiliations:** 1 Environmental Health Science and Research Bureau, Health Canada, Ottawa, Ontario, Canada; 2 Water and Air Quality Bureau, Health Canada, Ottawa, Ontario, Canada; 3 The Ottawa Hospital Research Institute, Ottawa, Ontario, Canada; 4 ICES uOttawa, Ottawa Hospital Research Institute, Ottawa, Ontario, Canada; 5 School of Epidemiology and Public Health, University of Ottawa, Ottawa, Ontario, Canada; 6 Dalla Lana School of Public Health, University of Toronto, Toronto, Ontario, Canada; 7 Department of Epidemiology, Biostatistics, and Occupational Health, McGill University, Montreal, Quebec, Canada; 8 Climate Change and Health Office, Health Canada, Ottawa, Ontario, Canada; 9 Children’s Health Research Institute, London, Ontario, Canada; 10 Schulich School of Medicine and Dentistry, Western University, London, Ontario, Canada; 11 Department of Energy, Environmental, and Chemical Engineering McKelvey School of Engineering, St Louis, Missouri; 12 Department of Geomatics, University of Sherbrooke, Sherbrooke, Canada

## Abstract

**Importance:**

Prenatal and early-life exposure to fine particulate matter (PM_2.5_) has been associated with autism spectrum disorder (ASD), but the role of individual components and timing of exposure remains unclear.

**Objective:**

To examine associations between prenatal and first-year-of-life exposure to PM_2.5_ components and ASD diagnosis, and identify potentially sensitive periods during pregnancy.

**Design, Setting, and Participants:**

This cohort study conducted in Ontario, Canada, used administrative health data covering approximately 98% of births in the province. The cohort included singleton live births from Ontario hospitals between April 1, 2002, and December 31, 2022, with gestational age 36 to 42 weeks, birth weight 500 to 6800 g, maternal age 15 to 55 years, complete residential history, and provincial health insurance coverage. At least 18 months of follow-up was required for postnatal exposure analyses.

**Exposures:**

Biweekly concentrations of PM_2.5_ components (black carbon, dust, ammonium, nitrate, organic matter, sulfate, sea salt) and weekly NO_2_ and O_3_ from conception to age 36 weeks. Prenatal models were adjusted for postnatal exposure using annual averages. Pollutant levels were estimated using satellite data, chemical transport models, and ground-based measurements.

**Main Outcomes and Measures:**

ASD diagnosis by age 5 years. Cox proportional hazards models were used to assess associations, and distributed lag nonlinear models identified sensitive exposure windows.

**Results:**

Among 2 183 324 births (mean [SD] maternal age, 30.5 [5.4] years; mean [SD] gestational age at birth, 39.2 [1.1] weeks; 1 152 040 female infants [48.9%]), prenatal exposure to PM_2.5_, sulfate (SO_4_^2−^), and ammonium (NH_4_^+^), and postnatal exposure to ozone (O_3_), were associated with ASD. Hazard ratios (HRs) per 1-IQR increase for SO_4_^2−^ were 1.15 (95% CI, 1.06-1.25) and for NH_4_^+^ was 1.12 (95% CI, 1.01-1.23). PM_2.5_ mass excluding SO_4_^2−^ and NH_4_^+^ during their respective critical windows was not associated with ASD (HR, 1.04; 95% CI, 0.92-1.19). O_3_ exposure during weeks 26 to 30 (HR, 1.03; 95% CI, 1.00-1.05) and over the first year (HR, 1.09; 95% CI, 1.01-1.17) was also associated with ASD.

**Conclusions and Relevance:**

In this large cohort study, prenatal exposure to specific PM_2.5_ components and postnatal O_3_ exposure were associated with ASD risk. The second and third trimesters may represent sensitive exposure windows. These findings support further research on air pollution’s role in ASD etiology.

## Key Points

**Question:**

Are prenatal and early-life exposures to fine particulate matter (PM_2.5_) and its components associated with increased risk of autism spectrum disorder (ASD) in childhood, and do certain gestational periods represent sensitive windows for these associations?

**Findings:**

In this cohort study of 2 183 324 births in Ontario, Canada, higher prenatal exposures to sulfate and ammonium components of PM_2.5_ were statistically associated with increased ASD risk. The second and third trimesters appeared to be sensitive exposure windows.

**Meaning:**

These findings suggest that exposure to sulfate and ammonium components of PM_2.5_ during the second and third trimesters are associated with increased ASD risk.

## Teaser

This cohort study of births in a Canadian province examines whether higher prenatal exposures to fine particulate matter and its components are associated with risk of autism spectrum disorder.

## Introduction

Fine particulate matter (PM_2.5_) is a well-established air pollutant linked to a wide range of adverse health outcomes.^1,2,3^ Despite regulatory advances,^4,5,6^ PM_2.5_ exposure remains a public health concern, particularly in urban areas with substantial industrial and vehicular emissions.^7^

Prenatal and early postnatal exposure to PM_2.5_ is of particular concern, as it has been associated with neurodevelopmental outcomes, including autism spectrum disorder (ASD).^8,9,10,11,12,13,14^ Clinical and animal studies suggest several biological pathways through which PM_2.5_ may influence neurodevelopment, including epigenetic modifications,^15,16^ pro-inflammatory responses,^16,17,18^ and oxidative stress.^16,19^ These mechanisms are thought to contribute to structural and functional changes in the developing brain.^20,21^ However, most epidemiological studies have focused on total PM_2.5_ mass, often overlooking potential variability in toxic effects among its chemical components.^22^

There is limited evidence regarding associations between ASD and nonmetal PM_2.5_ components, as well as the timing of these exposures. A large Southern California cohort study reported associations between prenatal exposure to elemental carbon, black carbon, organic matter, and sulfate (SO_4_^2−^), with stronger effect estimates for elemental carbon, black carbon, and organic matter during the first 2 trimesters, and SO_4_^2−^ during the third.^23^ A follow-up study from the same group also found an association with nitrate (NO_3_^−^).^24^ However, these studies did not consider whether early postnatal exposures might interact with prenatal exposures to further influence ASD risk.

Although some research has examined potential effect modifiers such as neighborhood income^25,26^ and racial or immigration status^10,14,26,27^ in relation to total PM_2.5_, less is known about how these factors may influence associations with individual chemical components. Moreover, evidence on other ambient pollutants such as nitrogen dioxide (NO_2_)^28,29,30,31^ and ozone (O_3_)^10,14,25,26,31^ remains limited and inconsistent.

To address these gaps, we conducted a population-based retrospective cohort study using linked administrative data from Ontario, Canada (2002-2022), to examine associations between prenatal and early-life exposure to PM_2.5_ components, NO_2_, and O_3_ with ASD diagnoses. We employed distributed lag nonlinear models to identify potentially sensitive exposure windows. Findings may inform source-specific regulations, refine environmental risk assessments, and guide early-life interventions.

## Methods

### Study Population and Data Sources

We identified all in-hospital singleton live births in Ontario from April 1, 2002, to December 31, 2022 (2 744 291 total records). Records were excluded as invalid or duplicate records (8730 records); no continuous Ontario Health Insurance Plan (OHIP) eligibility (92 504 records); maternal nonresidency or missing postal code (4286 records); multiple births (87 968 records); missing or invalid covariates, exposure estimates, or follow-up (160 023 records); follow-up occurring before 18 months (172 128 records); and missing gestational age or sex (35 328 records). The final cohort included 2 183 324 children (eFigure 1 in Supplement 1).

Maternal-infant linkages used the Registered Persons Database (RPDB) and the Mother-Baby Linked database (MOMBABY) (approximately 98% of births).^32^ Maternal smoking and body mass index (BMI) (2006-2021) were sourced from the Better Outcomes Registry and Network (BORN) for descriptive purposes, and chronic maternal conditions from administrative datasets.

Area-level socioeconomic status was assessed via the Ontario Marginalization Index (ON-Marg) (eMethods in Supplement 1),^33^ and residential history via Statistics Canada’s Postal Code Conversion File (PCCF). Outcome data were sourced from the Canadian Institute for Health Information Discharge Abstract Database (CIHI-DAD), the National Ambulatory Care Reporting System (NACRS), and OHIP Records Database. Linkages were performed at ICES using encoded identifiers.

This study followed the Strengthening the Reporting of Observational Studies in Epidemiology (STROBE) reporting guideline and was approved by the Health Canada Research Ethics Board. Informed consent was waived due to minimal risk.

### Exposure Assessment

Prenatal exposures were assigned by maternal postal code at delivery, using geographic centroids. Weekly average NO_2_ and O_3_ and biweekly PM_2.5_ (total mass and components) were estimated from conception to age 36 weeks, and first-year postnatal exposures as annual postal code–level averages, weighted by duration at each address.

PM_2.5_ mass and component concentrations were obtained from established satellite and ground-based models by the Atmospheric Composition Analysis Group at Washington University in St Louis (eMethods in Supplement 1).^34^ Components included black carbon, dust, ammonium (NH_4_^+^), nitrate (NO_3_^−^), organic matter, sulfate (SO_4_^2−^), and sea salt. Cross-validation *R^2^* ranged from 0.78-0.88 for sulfate, nitrate, and ammonium, and 0.43-0.67 for others.^34^

Weekly NO_2_ (2002-2022) was modeled via national land-use regression (LUR) using NAPS data, satellite-derived 2020 estimates, road lengths, industrial land use, and mean summer rainfall, with spatiotemporal interpolation from the Canadian Urban Environmental Health Research Consortium (CANUE).^35,36^ O_3_ was estimated on a 21-km grid of daily maximum concentrations during the warm season (May 1 to October 31), with weekly values similarly derived using CANUE and NAPS data.^35,37^

### Outcome Ascertainment

Follow-up began at birth and ended at ASD diagnosis, loss to follow-up, age 5 years, or December 31, 2022. ASD was identified using a validated algorithm requiring 1 or more inpatient F84.x *International Statistical Classification of Diseases and Related Health Problems, Tenth Revision (ICD-10)* code or 3 or more outpatient OHIP 299 codes spaced 2 or more weeks over 3 years.^38,39,40^ Sensitivity, specificity, positive predictive value, and negative predictive value were 50.0% (95% CI, 40.7%-88.7%), 99.6% (95% CI, 99.4%-99.7%), 56.6% (95% CI, 46.8%-66.3%), and 99.4% (95% CI, 99.3%-99.6%), respectively.^39^

### Covariates

A priori covariates included infant sex, birth weight, maternal parity, prepregnancy hypertension or diabetes, season of birth (spring, summer, fall, winter), maternal age (modeled with natural splines, 3 degrees of freedom [*df*]), ON-Marg domains, neighborhood income quintile, rurality, Greater Toronto Area residence, and public health region. Birth year and calendar week (1 to 52) were modeled separately using natural splines (3 *df*). Adjustments were informed by a directed acyclic graph (DAG) generated using DAGitty (version 3.1) (eFigure 2 in the Supplement).^24,41,42,43^

### Statistical Analyses

Population characteristics and pollutant concentrations were summarized; continuous variables as means (with SD) and categorical as frequencies (with percentages). Pearson correlations were computed for prenatal and first-year pollutant averages.

Cox proportional hazards models estimated associations between air pollution and ASD risk, reporting hazard ratios (HRs) and 95% CIs per 1-IQR increase. PM_2.5_ components were analyzed using 3 approaches: (1) single-pollutant models; (2) PM_2.5_ residual-adjusted models (ie, PM_2.5_ for approximately each component)^22^; and (3) early-life exposure-adjusted models, incorporating residual PM_2.5_ and first-year exposure. Models were adjusted for DAG-identified confounders.

Sensitivity analysis addressed spatial confounding with 2-level mixed-effects Cox models with random intercepts for census divisions (analogous to counties) and either census tracts (urban areas) or census subdivisions (rural municipalities).^44,45^ Owing to computational limitations, these models were restricted to the primary single-pollutant estimates for PM_2.5_ and its components. Distributed lag nonlinear models (DLNMs) identified sensitive prenatal exposure windows by modeling nonlinear exposure-response relationships.^46^ For identified sensitive windows, we performed concentration-response analyses.

Joint PM_2.5_ component associations were estimated with quantile-based g-computation, restricted to components associated with ASD risk in the early-life exposure-adjusted models. Spline degrees of freedom for pollutant concentrations were selected based on the Akaike information criterion (AIC).^47^ Models with 3 *df* for black carbon, dust, sea salt, and O_3_; 4 *df* for NO_2_; and 5 *df* for PM_2.5_, NO_3_^−^, NH_4_^+^, OM, and SO_4_^2−^. Sensitive exposure windows were where HR above 1.00 and 95% CI excluded 1.00. Effect modification was tested via multiplicative interaction terms for sex, maternal asthma, neighborhood income quintile, proportion of racialized or newcomer populations, and rurality, with stratum-specific estimates. Interaction was considered significant at *P* < .05.

Analyses used R version 3.6.1 (R Project for Statistical Computing); DLNMs analyses were performed using the dlnm package version 2.4.7. All tests were 2-sided, and *P* < .05 was considered statistically significant. Proportional hazards assumption were tested with Schoenfield residuals; no violations were observed.

## Results

### Descriptive Statistics

We included 2 183 324 singleton live births, with a mean (SD) maternal age at delivery of 30.5 (5.4) years and a mean (SD) gestational age of 39.2 (1.1) weeks. Among the cohort, 1 203 412 (51.1%) were boys and 1 152 040 (48.9%) were girls. A total of 19 569 children (0.8%) were diagnosed with ASD before age 5 years, including 15 205 boys and 4364 girls; ASD was approximately 3.5 times more commonly diagnosed among boys. Demographic and socioeconomic characteristics of the study participants are summarized in Table. Children diagnosed with ASD were more frequently observed among those born to nulliparous mothers and mothers with underlying health conditions, including asthma, diabetes, and hypertension. They were also more likely to reside in large urban areas (population 1.5 million or more) and neighborhoods with lower income, higher ethnic concentration, greater material deprivation, and poorer housing conditions.

**Table.** Demographic and Socioeconomic Characteristics of Study Participants

Characteristics	Births, No. (%)	
	Total cohort (N = 2 355 452)	ASD (n = 19 569)
**Individual-level characteristics**		
Maternal age at delivery, mean (SD), y	30.5 (5.4)	30.8 (5.6)
Maternal prepregnancy BMI		
<18.5 (Underweight)	46 382 (2.0)	644 (3.3)
18.5-24.9 (Healthy weight)	484 954 (20.6)	5110 (26.1)
25.0-29.9 (Overweight)	237 549 (10.1)	2802 (14.3)
≥30.0 (Obesity)	189 988 (8.1)	2626 (13.4)
Unknown	1 396 541 (59.3)	8385 (42.9)
Maternal preexisting health condition		
Prepregnancy asthma	336 566 (14.3)	3151 (16.1)
Prepregnancy cardiovascular disease	29 (<0.1)	≤5 (<0.1)
Prepregnancy diabetes^a^	31 409 (1.3)	438 (2.2)
Prepregnancy hypertension	48 863 (2.1)	597 (3.1)
Maternal smoking during pregnancy		
Yes	164 233 (7.0)	2068 (10.6)
No	1 497 609 (63.6)	15 378 (78.6)
Unknown	693 610 (29.5)	2123 (10.9)
Follow-up time after birth, mean (SD), y	4.4 (1.3)	2.8 (0.8)
Infant sex		
Male	1 203 412 (51.1)	15 205 (77.7)
Female	1 152 040 (48.9)	4364 (22.3)
Gestational age, mean (SD), wk	39.2 (1.1)	39.1 (1.2)
Parity		
0	1 021 739 (43.4)	9881 (50.5)
1	867 586 (36.8)	6278 (32.1)
≥2	466 127 (19.8)	3410 (17.4)
Birth weight, mean (SD), g	3442.1 (473.4)	3415.9 (495.0)
Season of birth		
Spring	589 789 (25.0)	4692 (24.0)
Summer	630 418 (26.8)	5123 (26.2)
Fall	601 458 (25.5)	5177 (26.5)
Winter	533 787 (22.7)	4577 (23.4)
**Neighborhood-level characteristics**		
Residence at birth		
Urban	2 113 324 (89.7)	18 422 (94.1)
Rural	240 446 (10.2)	1119 (5.7)
Unknown	1682 (0.1)	28 (0.1)
Community size		
≥1 500 000	1 068 416 (45.4)	11 286 (57.7)
500 000-1 499 999	351 310 (14.9)	2226 (11.4)
100 000-499 999	519 226 (22.0)	3764 (19.2)
10 000-99 999	172 826 (7.3)	1142 (5.8)
<10 000	240 440 (10.2)	1118 (5.7)
Unknown	3234 (0.1)	33 (0.2)
**Quintile results^b^**		
Neighborhood income		
First	516 308 (21.9)	5791 (29.6)
Second	475 277 (20.2)	4251 (21.7)
Third	484 665 (20.6)	3837 (19.6)
Fourth	483 784 (20.5)	3398 (17.4)
Fifth	386 800 (16.4)	2238 (11.4)
Unknown	8618 (0.4)	54 (0.3)
Household and dwelling^c^		
First	489 046 (20.8)	3937 (20.1)
Second	436 429 (18.5)	3061 (15.6)
Third	420 891 (17.9)	3095 (15.8)
Fourth	445 706 (18.9)	3682 (18.8)
Fifth	531 339 (22.6)	5664 (28.9)
Unknown	32 041 (1.4)	130 (0.7)
Material resources^d^		
First	469 449 (19.9)	2828 (14.5)
Second	446 590 (19.0)	3168 (16.2)
Third	440 555 (18.7)	3405 (17.4)
Fourth	442 418 (18.8)	4111 (21.0)
Fifth	524 399 (22.3)	5927 (30.3)
Unknown	32 041 (1.4)	130 (0.7)
Age and labor force^e^		
First	760 441 (32.3)	7000 (35.8)
Second	502 421 (21.3)	4332 (22.1)
Third	405 184 (17.2)	3253 (16.6)
Fourth	351 728 (14.9)	2598 (13.3)
Fifth	303 637 (12.9)	2256 (11.5)
Unknown	32 041 (1.4)	130 (0.7)
Racial minority and newcomer populations^f^		
First	309 281 (13.1)	1705 (8.7)
Second	349 133 (14.8)	2079 (10.6)
Third	391 847 (16.6)	2785 (14.2)
Fourth	497 109 (21.1)	4086 (20.9)
Fifth	776 041 (33.0)	8784 (44.9)
Unknown	32 041 (1.4)	130 (0.7)
Particle levels during pregnancy, mean (SD)		
PM_2,5_, μg/m^3^	7.8 (2.0)	7.5 (1.7)
NO_2_, ppb	5.7 (3.0)	5.8 (3.2)
O_3_, ppb	46.3 (9.6)	45.3 (9.4)
Particle levels during childhood, mean (SD)		
PM_2,5_, μg/m^3^	7.7 (1.9)	7.2 (1.6)
NO_2_, ppb	5.7 (2.8)	5.8 (3.0)
O_3_, ppb	46.2 (9.2)	45.0 (9.1)

Abbreviations: ASD, autism spectrum disorder; BMI, body mass index (calculated as weight in kilograms divided by height in meters squared); NO_3_, nitrate; O_3_, ozone; PM_2.5_, fine particulate matter (with a diameter ≤2.5 μm); ppb, parts per billion.

^a^ Type 1 and type 2 diabetes diagnosed before pregnancy.

^b^ Quintiles were obtained from the Ontario Marginalization Index. For each census year and each marginalization dimension, postal codes were scored, ranked, and categorized into quintiles, with quintile 1 representing the lowest degree of marginalization and quintile 5 representing the greatest degree of marginalization.

^c^ Households and dwellings includes indicators of residential and family stability (eg, percentage living alone, percentage of dwellings not owned).

^d^ Material resources include markers of socioeconomic disadvantage (eg, percentage unemployed, percentage without a high school diploma).

^e^ Age and labor force includes demographic and workforce characteristics (eg, percentage aged ≥65 years, dependency ratio, percentage not in the labor force).

^f^ Racial minority and newcomer populations is the proportion of recent immigrants and individuals identifying as visible minorities (as defined by Statistics Canada).

### Air Pollution Exposure

The mean (SD) prenatal PM_2.5_ concentration was 7.8 (2.0) μg/m^3^, with a slight decrease in the first year of life (7.7 [1.9] μg/m^3^). Mean (SD) prenatal concentrations of NO_2_ (5.7 [3.0] parts per billion [ppb]) and O_3_ (46.3 [9.6] ppb) were comparable during childhood. Detailed exposure data, including PM_2.5_ components, are available in eTable 1 in the Supplement 1. Prenatal PM_2.5_ was strongly correlated with black carbon (BC) (*r* = 0.75), ammonium (NH_4_^+^) (*r* = 0.81), and sulfate (SO_4_^2−^) (*r* = 0.78), whereas correlations with NO_2_ (r = 0.17) and O_3_ (*r* = −0.11) were weak (eTable 2 in Supplement 1) and remained weak during the first year of life (NO_2_: *r* = 0.22; O_3_: *r* = −0.11) (eTable 3 in Supplement 1). Prenatal pollutant concentrations were also strongly correlated with exposure during the first year of life (eTable 4 in Supplement 1).

### Associations Between ASD and Prenatal Air Pollution

**Figure 1.**
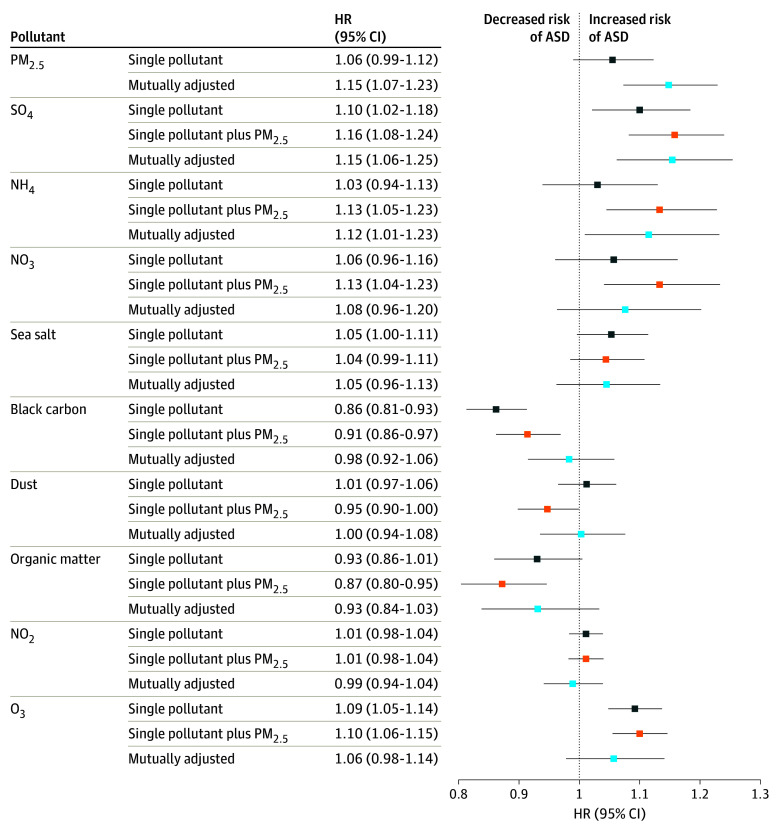
Cumulative Hazard Ratios (HRs) for the Association Between Prenatal Outdoor PM_2.5_ Mass and Component Concentrations Over the Entire Pregnancy All models were adjusted for infant sex, birth weight, maternal parity, maternal prepregnancy history of hypertension and diabetes (1 or more diagnosis), season of birth (spring, summer, fall, winter), maternal age, area-level socioeconomic status variables, urbanicity (urban vs rural), geographic indicators, birth year, and calendar week in a year of birth. Fine particulate matter (PM_2.5_) residual variations-adjusted models included all variables in single-pollutant models plus the PM_2.5_ residuals (ie, PM_2.5_ of each component) for models for single components or PM_2.5_ total mass for models for nitrogen dioxide (NO_2_) and ozone (O_3_). Early life exposure-adjusted models included all variables from the previous models plus exposure to PM_2.5_ and the selected pollutant during the first year of life. ASD indicates autism spectrum disorder; NH_4_, ammonium; NO_3_, nitrate; SO_4_, sulfate.

**Figure 2.**
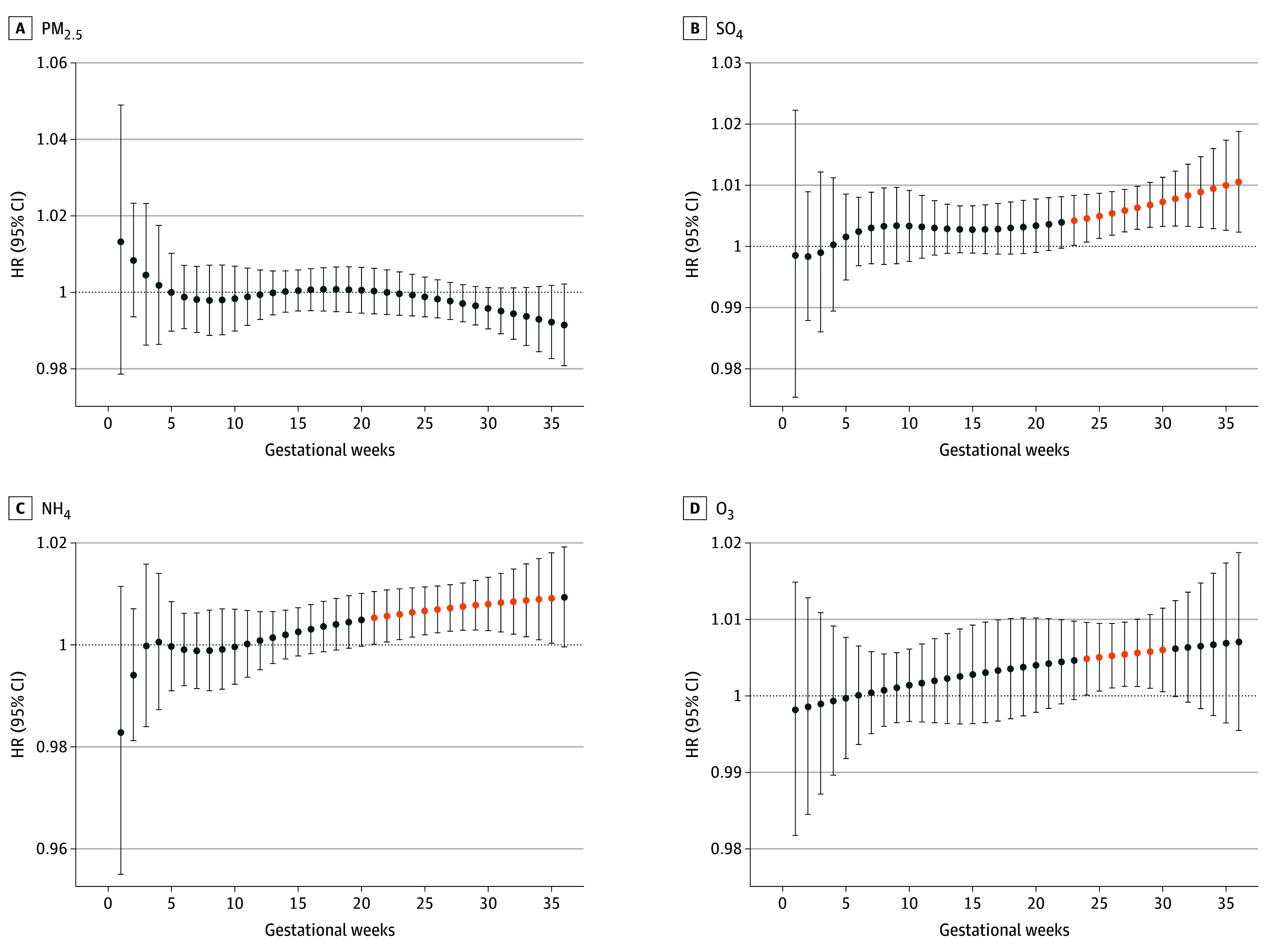
Weekly Hazard Ratios (HRs) for Autism Spectrum Disorder and PM_2.5_, SO_4_, NH_4_, and O_3_ Concentrations Over the Gestational Period Lines represent 95% CIs, with orange points indicating gestational weeks for which the HR was statistically significant (ie, not comprising the null value). All models were adjusted for infant sex, birth weight, maternal parity, maternal prepregnancy history of hypertension and diabetes (1 or more diagnosis), season of birth (spring, summer, fall, winter), maternal age, area-level socioeconomic status variables, urbanicity (urban vs rural), geographic indicators, birth year, and calendar week in a year of birth. HR for the distributed lag model-identified sensitive window for sulfate (SO_4_) from 23 to 36 weeks of gestation is 1.11 (1.04-1.18). HR for the Distributed Lag Model-identified sensitive window for ammonium (NH_4_) from 21 to 34 weeks of gestation 1.11 (1.04-1.19). HR for the Distributed Lag Model-identified sensitive window for ozone (O_3_) from 22 to 30 weeks of gestation is 1.03 (1.00-1.05). PM_2.5_ indicates fine particulate matter.

After adjusting for PM_2.5_ residuals and first-year exposure, significant associations remained for NH_4_^+^ and SO_4_^2−^, while associations for other pollutants were attenuated (Figure 1). Prenatal PM_2.5_ exposure was significantly associated with increased ASD risk when adjusted for the average exposure during the first year of life (HR, 1.15; 95% CI, 1.07-1.23; per 1-IQR increase, 3.5 μg/m^3^ increase). However, this association was no longer significant after adjusting for NH_4_^+^ and SO_4_^2−^ during their respective critical windows (HR, 1.04; 95% CI, 0.916-1.19) (Figure 2). In contrast, average first-year-of-life exposure to PM_2.5_ was negatively associated with ASD risk (HR, 0.87; 95% CI, 0.84-0.91). SO_4_^2−^ exposure showed consistent associations across all models (eg, HR, 1.15; 95% CI, 1.06-1.25; per 1-IQR increase, 0.95 μg/m^3^ increase). Prenatal NH_4_^+^ exposure was significantly associated with ASD risk in PM_2.5_ residual variations-adjusted (HR, 1.13; 95% CI, 1.05-1.23, per 1-IQR increase, 0.60 μg/m^3^ increase) and postnatal exposure-adjusted models (HR, 1.12; 95% CI, 1.01-1.23). Exposure to O_3_ was positively associated with ASD in PM_2.5_ residual variations-adjusted models (HR, 1.10; 95% CI, 1.06-1.15; per 1-IQR increase, 19.13 ppb increase), but this association was attenuated after adjusting for first-year exposure. Notably, O_3_ was the only pollutant for which first-year exposure remained significantly associated with ASD risk (HR, 1.09; 95% CI, 1.01-1.17) (eTable 5 in Supplement 1). A quartile increase in the mixture of prenatal NO_3_^−^ and SO_4_^2−^, and first-year NH_4_^+^ and SO_4_^2−^, resulted in a small increase in ASD risk that was not significant (HR, 1.02; 95% CI, 0.96-1.08). Estimated contributions to this association were 7.2%, 2.6%, 38.3%, and 51.9% for prenatal NO_3_^−^, SO_4_^2−^, and first-year NH_4_^+^, SO_4_^2−^, respectively (eTable 6 in Supplement 1). We observed slightly attenuated effect sizes for the association between prenatal pollutant exposure and ASD risk when including 2 levels of random intercepts for spatial clusters in the models (eTable 7 in Supplement 1). Therefore, we pursued the main analyses without including these random intercepts. We also reported the associations between PM_2.5_ with ASD risk using different degrees of freedom in the distributed lags and found that the HRs were similar from 3 *df* to 5 *df* (eTable 8 in Supplement 1).

### Sensitive Windows of Exposure

Sensitive gestational windows associated with ASD risk were identified using postnatal-exposure–adjusted models (Figure 2). Statistically significant associations were observed for PM_2.5_ exposure during weeks 14 to 32 (HR, 1.12; 95% CI, 1.07-1.18), SO_4_^2−^ during weeks 23 to 36 (HR, 1.11; 95% CI, 1.04-1.18), NH_4_^+^ during weeks 21 to 34 (HR, 1.11; 95% CI, 1.04-1.19), and O_3_ during weeks 26 to 30 (HR, 1.03; 95% CI, 1.00-1.05).

### Concentration-Response Analysis

**Figure 3.**
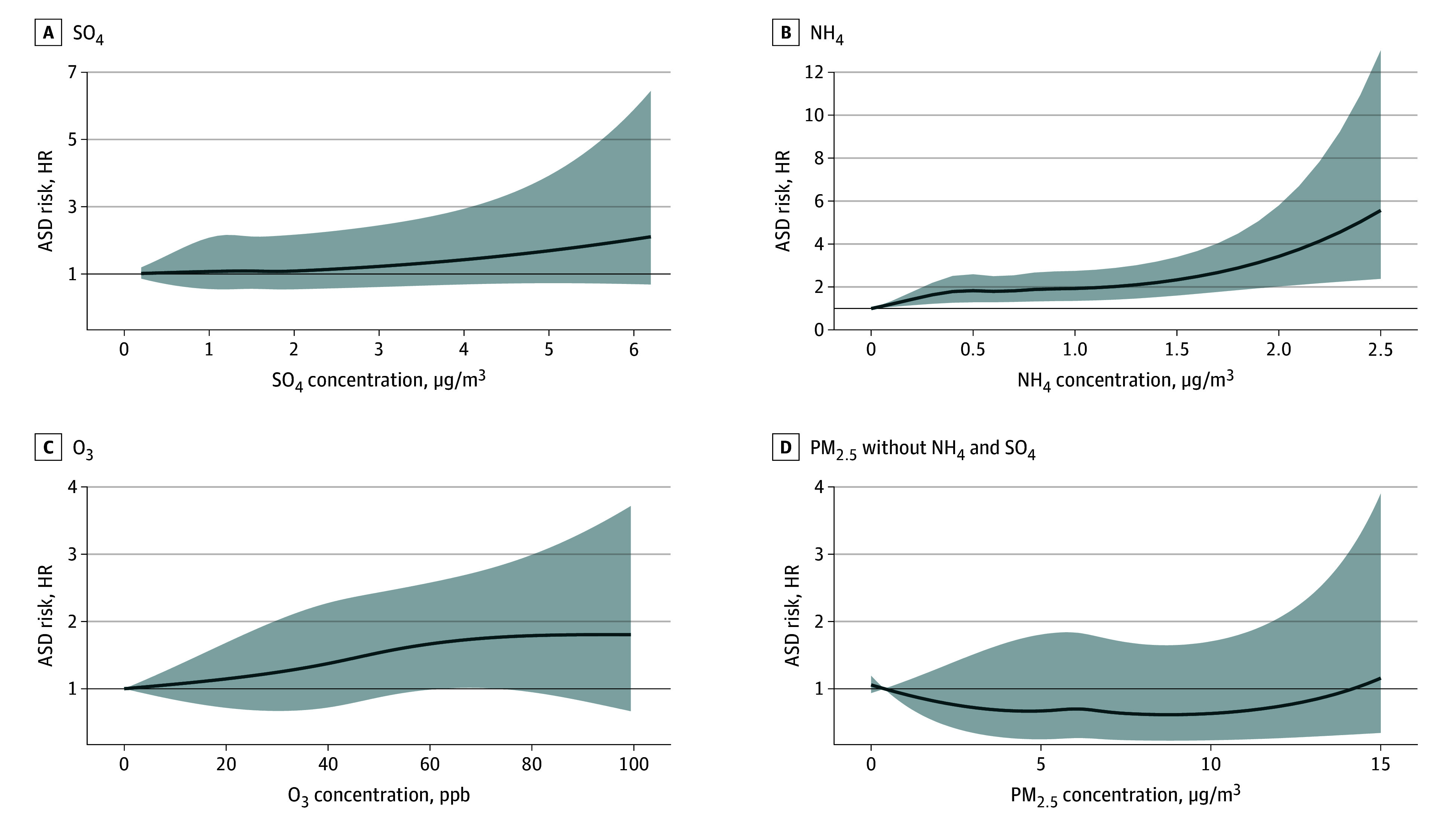
Concentration-Response Function for the Associations Between SO_4_, NH_4_, O_3_, and PM_2.5_ Mass Minus the Average NH_4_ and SO_4_ With ASD Shaded areas indicate 95% confidence intervals. All models were adjusted for infant sex, birth weight, maternal parity, maternal prepregnancy history of hypertension and diabetes (1 or more diagnosis), season of birth (spring, summer, fall, winter), maternal age, area-level socioeconomic status variables, urbanicity (urban vs rural), geographic indicators, birth year, and calendar week in a year of birth. HR for the Distributed Lag Model-identified sensitive window for sulfate (SO_4_) from 23 to 36 weeks of gestation is 1.11 (1.04-1.18). HR for the Distributed Lag Model-identified sensitive window for ammonium (NH_4_) from 21 to 34 weeks of gestation 1.11 (1.04-1.19). HR for the distributed lag model-identified sensitive window for ozone (O_3_) from 22 to 30 weeks of gestation is 1.028 (1.00-1.05). ASD indicates autism spectrum disorder; PM_2.5_, fine particulate matter.

Distinct nonlinear exposure-response patterns were observed for pollutants (Figure 3). For PM_2.5_, the estimated ASD risk initially decreased at low concentrations, reaching a minimum around 5 μg/m^3^, before stabilizing and then rising sharply beyond 10 μg/m^3^. For SO_4_^2−^, the estimated ASD risk increased steeply at concentrations above 2 μg/m^3^, with a pronounced rise at 5 μg/m^3^. For NH_4_^+^, estimated risk remained stable at concentrations below 1.5 μg/m^3^ but rose sharply at 2 μg/m^3^. For O_3_, estimated ASD risk increased with concentrations up to 60 ppb, plateauing beyond this level.

### Stratified Analyses

In urban settings, estimated effect sizes for associations were larger for PM_2.5_ (HR, 1.09; 95% CI, 1.05-1.14; *P* < .001), SO_4_^2−^ (HR, 1.15; 95% CI, 1.10-1.21; *P* value = .03), and NH_4_^+^ (HR, 1.16; 95% CI, 1.09-1.23; *P* = .02) compared with rural areas (PM_2.5_: HR, 1.104; 95% CI, 0.939-1.299; SO_4_^2−^: HR, 1.102; 95% CI, 0.921-1.317; NH_4_^+^: HR, 1.269; 95% CI, 0.984-1.639) (eTable 9 in Supplement 1). Sex-stratified analyses revealed larger estimated effect sizes for the association with all pollutants in male than in female children, with SO_4_^2−^ being the only pollutant significantly associated with ASD risk in female children (eTable 10 in Supplement 1). The association with PM_2.5_, SO_4_^2−^, and NH_4_^+^ were statistically significant in children of both asthmatic and nonasthmatic mothers, although no statistically significant effect modification was observed. For O_3_, a statistically significant positive association with ASD was observed, which appeared more pronounced among children born to asthmatic mothers (HR, 1.064; 95% CI, 0.947-1.196) compared with the rest of the cohort (HR, 1.037; 95% CI, 0.984-1.092; *P* = .02) (eTable 11 in Supplement 1). Elevated estimated increased risk for ASD associated with increased PM_2.5_, SO_4_^2−^ and NH_4_^+^ were more pronounced in lower-income and middle-income neighborhoods (eTable 12 in Supplement 1). Additionally, neighborhoods with higher concentrations of racialized and newcomer populations exhibited larger effects sizes for the association of ASD risk with these pollutants (eTable 13 in Supplement 1).

## Discussion

In this large population-based cohort from Ontario, Canada, we observed significant associations between prenatal PM_2.5_ exposure, particularly its components SO_4_^2−^ and NH_4_^+^, and increased ASD risk in children. The effect size of this association was largest during the second and third trimesters, suggesting critical windows of vulnerability. Additionally, O_3_ exposure in late pregnancy and the first postnatal year was significantly associated with ASD. Stratified analyses further suggested that sociodemographic factors may modify these associations.

### Air Pollution and Risk of ASD

Our study adds evidence showing an association of prenatal PM_2.5_ exposure, particularly during mid to late pregnancy, with increased ASD risk. HRs for pregnancy-average PM_2.5_ were consistent with most previous studies.^11,12,14,23,24,26,28,48,49^

We noted lower average PM_2.5_ concentrations during the first postnatal year than during pregnancy, likely reflecting a temporal decline in ambient PM_2.5_ in Ontario from 2002 to 2022.^50^ Although mechanisms remain unclear, oxidative stress and neuroinflammation are plausible pathways linking PM_2.5_ exposure to ASD.^51,52,53,54,55,56^ PM_2.5_ has also been associated with gut microbiota dysbiosis, which may trigger systemic inflammation and disrupt tryptophan metabolism, potentially contributing to neurodevelopmental disorders via the gut-brain axis.^57^ Epigenetic changes, such as DNA methylation of ASD-related genes,^58^ also represent plausible mechanisms for PM_2.5_ neurotoxicity.

### SO_4_^2−^ and NH_4_^+^: Potential Contributors to PM_2.5_ and ASD Risk

We identified SO_4_^2−^ and NH_4_^+^, 2 major PM_2.5_ components, as having the highest risk in the association of prenatal air pollution with ASD risk. These associations remained significant after adjusting for residual PM_2.5_ and first-year exposure, suggesting SO_4_^2−^ and NH_4_^+^ may partly drive the PM_2.5_-ASD association.^11,12,14,26,28,48,49^ However, they may also serve as proxies for other co-emitted pollutants such as metals from sulfur dioxide (SO_2_) emissions related to coal combustion.^59,60^

Recent studies,^23,24^ including a large 2022 cohort study from Southern California,^23^ have reported similar prenatal SO_4_^2−^ associations with ASD, with the highest risk during the third trimester (HR, 1.13; 95% CI, 1.06-1.20; per 1-IQR increase, 0.50 μg/m^3^), consistent with our results. While epidemiological evidence linking NH_4_^+^ to ASD is scarce, laboratory studies suggest it may affect neurodevelopment by disrupting astrocytes^61^ and neuronal neurotransmission.^62^

### Mid to Late Pregnancy and Early Postnatal Period: Critical Developmental Windows

We identified the second and third trimesters, particularly weeks 14 to 32, as periods when prenatal PM_2.5_ exposure had the highest risk of ASD, with narrower windows for SO_4_^2−^ (weeks 23 to 36) and NH_4_^+^ (weeks 21 to 34). These findings align with prior cohort and case-control studies showing increased risk in late pregnancy.^11,23^

While prenatal exposures yielded larger effect estimates in single pollutant and first-year–adjusted models, postnatal SO_4_^2−^ and NH_4_^+^ exposures contributed more to the overall PM_2.5_-ASD association in quantile g-computation. Late pregnancy is critical for placental function, fetal growth, and brain development,^63,64,65^ including the myelination, neuronal organization, and synaptogenesis,^66^ potentially increasing vulnerability to pollutants. First-year exposures cover a longer, more heterogeneous period, possibly explaining their stronger contribution in mixture analyses. Correlation between prenatal and postnatal exposures may also attenuate associations when both periods are modeled. Early pregnancy^26,49^ and postnatal exposure^12,14,67,68,69,70^ may also represent susceptibility windows. Limited epidemiologic evidence on SO_4_^2−^ and NH_4_^+^ highlights the need for further investigation into their neurotoxic effects.^23,24^

First-year O_3_ exposure was also associated with ASD, consistent with findings from a study in metropolitan Cincinnati.^14^ Early postnatal brain development involves rapid maturation, oligodendrocyte proliferation, myelination onset, and synaptic pruning.^71,72^ O_3_ exposure during this time has been linked to oxidative stress and mitochondrial dysfunction,^73^ and animal models have shown ASD-like behaviors.^74^ In our study, postnatal rather than prenatal O_3_ exposure remained significantly associated with ASD risk after adjusting for the other period and PM_2.5_.

### Patterns by Sociodemographic and Other Factors

Stratified analyses showed PM_2.5_ exposure in urban, but not rural, areas was associated with higher ASD risk, especially in low-income and middle-income neighborhoods with a high proportion of racial minority or newcomer populations. These patterns suggest environmental injustice.

In multipollutant models, SO_4_^2−^ and NH_4_^+^ emerged as key contributors to the observed association between PM_2.5_ and ASD. SO_4_^2−^ is primarily linked to regional combustion sources such as coal-fired power plants,^75,76^ oil refineries,^75,76^ and marine engines using high-sulfur fuel,^77,78,79^ whereas NH_4_^+^ arises from sources including fertilizer use and sewage treatment,^76^ but is often correlated with tailpipe emissions from motor vehicles in urban areas.^80,81^ Both may indicate co-emitted toxicants, such as trace metals or acidic gases,^82^ associated with neuroinflammation and oxidative stress pathways relevant to ASD.^83^ Uneven pollution distribution may worsen disparities, as marginalized populations often live near high-emitting facilities or major highways.^84,85^ These findings highlight the need for source-specific research and for prioritizing interventions in high-risk areas.

### O_3_ and Asthma: Potential Relevance to Neurodevelopmental Outcomes

Although our findings suggest a potential positive association between prenatal O_3_ exposure in asthmatic mothers and ASD risk in children, the finding was not statistically significant and should be interpreted cautiously. Maternal asthma may increase fetal susceptibility to environmental exposures through immune-mediated mechanisms, as chronic systemic inflammation during pregnancy has been shown to disrupt the intrauterine environment and alter neurodevelopment.^86^

Epidemiologic studies have reported higher ASD risk among children of mothers with asthma,^87,88,89,90,91,92,93^ particularly when exacerbations occur in the first or second trimester.^90^ A meta-analysis reported a significant association with a modest effect (OR, 1.36; 95% CI, 1.28-1.44) with a higher risk among male children.^94^ Animal models of maternal allergic asthma (MAA) show that repeated allergen exposure during gestation elevates fetal brain cytokines (interleukin [IL]-6, IL-1α, interferon γ, granulocyte-macrophage colony-stimulating factor, TNFα), potentially altering microglial gene expression and producing ASD-like behavioral phenotypes.^95,96,97,98^ These findings may primarily involve the hippocampus and frontal cortex.^99^

O_3_ exposure may further amplify this susceptibility. Even below regulatory limits, O_3_ has been linked to airway inflammation, asthma exacerbation, and impaired lung function.^100,101,102,103^ In Quebec, prenatal O_3_ exposure was associated with childhood asthma (OR, 1.11; 95% CI, 1.10-1.12),^101^ despite average exposures below World Health Organization guideline limits.^104^ The potential for maternal asthma to modify the O_3_-ASD association merits further investigation.

### Study Strengths and Limitations

This study has several strengths, including its large cohort (over 2 million mother-infant pairs) and long period (2002-2022), which enhance statistical power and generalizability. The use of distributed lag models allowed detailed exploration of exposure timing and intensity.

Several limitations should be noted. Individuals excluded due to OHIP ineligibility, missing birth registry linkage, or incomplete follow-up could not be reliably characterized. Potential selection bias cannot be ruled out, and differences in socioeconomic status or other unmeasured factors may limit generalizability.

Although exposure estimates were assigned biweekly and linked to residential postal codes, they may not fully capture individual-level variability. Postal code–level assignment can introduce spatial misclassification, particularly in rural areas. Our estimates also did not account for time-activity patterns, daily mobility, maternal physical activity, housing, or air filtration, which can influence personal exposure.

Observed associations for SO_4_^2−^ and NH_4_^+^ may partly reflect reduced measurement error relative to other components. Satellite-based PM_2.5_ composition estimates showed highest agreement for SO_4_^2−^, NH_4_^+^, and NO_3_^−^ (*R^2^* = 0.75-0.86), and lower agreement for others (*R^2^* = 0.42-0.62), increasing power to detect significant associations.

Outcome ascertainment was based on a validated case-finding algorithm. Although it performed best among more than 150 tested algorithms,^105^ sensitivity was approximately 50% and positive predictive value 56.6%. The algorithm underidentifies younger children, female children, and higher-functioning ASD diagnosed in educational or community settings, and excludes nonphysician diagnoses. Misclassification is likely nondifferential, plausibly biasing effect estimates toward the null.

Exposure assessment was based on maternal residential address at birth, the most consistent and relevant location available for the cohort. Although the RPDB captures address changes semi-annually, it does not reliably reflect moves during pregnancy.

Finally, we could not account for potential interactions between air pollutants or individual-level factors such as race, ethnicity, lifestyle, differential health care access, and behavioral patterns, which may confound or modify associations between prenatal PM_2.5_ exposure and ASD risk. Further studies with richer individual-level data would be helpful to clarify observed patterns.

## Conclusions

In this population-based retrospective cohort study of 2 183 324 births in Ontario, Canada, we observed significant associations between prenatal air pollution, particularly PM_2.5_ components (SO_4_^2−^ and NH_4_^+^), and ASD risk, with the largest increase in risk during the second and third trimesters. Postnatal O_3_ exposure was also associated with elevated ASD risk, suggesting the early postnatal period may be an additional window of susceptibility. These findings underscore the potential importance of early-life environmental exposures and reinforce the need for public health strategies to reduce air pollution, particularly in urban and socioeconomically disadvantaged communities.

## Notes

Supplement 1. eMethods. eFigure 1. Flowchart of the inclusion and exclusion of the study population eFigure 2. Directed acyclic graph (DAG) for estimating the effect of ambient air pollution on ASD in children eTable 1. Average prenatal concentrations of ambient PM_2.5_, its components (BC, dust, NH_4_^+^, NO_3_^-^ , OM, SO_4_^2-^, SS), NO_2_, and O_3_ across childhood and pregnancy, and per trimester by ASD status eTable 2. Pearson correlation coefficients between the average prenatal concentrations of PM_2.5_, its components (BC, dust, NH_4_^+^, NO_3_^-^ , OM, SO_4_^2-^, SS), NO_2_, and O_3_ eTable 3. Pearson correlation coefficients between the average first-year-of-life concentrations of PM_2.5_, its components (BC, dust, NH_4_^+^, NO_3_^-^ , OM, SO_4_^2-^, SS), NO_2_, and O_3_ eTable 4. Pearson correlation coefficients between the average prenatal exposure and first year of life concentrations of PM_2.5_, NO_2_, and O_3_ eTable 5. Cumulative hazard ratios (HR) and 95% confidence intervals (CIs) describing the association between prenatal outdoor PM_2.5_ mass and component concentrations, NO_2_ and O_3_ with ASD in single-pollutant models, PM_2.5_ residual variations-adjusted models, and postnatal exposure-adjusted models over the entire pregnancy and first-year-of-life periods and Distributed Lag Model-identified sensitive windows eTable 6. Association between exposure to average prenatal and first-year-of-life NH_4_^+^, NO_3_^-^ and SO_4_^2-^ and ASD estimated by quantile-based g computation eTable 7. Cumulative hazard ratios (HR) and 95% confidence intervals (CIs) describing the association between prenatal outdoor PM_2.5_ mass and component concentrations, NO_2_ and O_3_ with ASD in single-pollutant models^a^ over the entire pregnancy using mixed effect models with two levels of spatial clusters as random effects: census division (equivalent in size to a county) and census tract within census divisions eTable 8. Cumulative hazard ratios (HR) and 95% confidence intervals (CIs) describing the association between prenatal outdoor PM_2.5_ mass with ASD in single-pollutant models^a^ over the entire pregnancy using different specifications of degrees of freedom in the distributed lag models over gestational weeks eTable 9. Hazard ratios (HRs) and 95% confidence intervals (CIs) for the associations between exposure to air pollutants and ASD stratified by rural/urban residence at birth eTable 10. Hazard ratios (HRs) and 95% confidence intervals (CIs) for the associations between exposure to air pollutants and ASD stratified by infant sex eTable 11. Hazard ratios (HRs) and 95% confidence intervals (CIs) for the associations between exposure to air pollutants and ASD stratified by maternal pre-existing asthma status eTable 12. Hazard ratios (HRs) and 95% confidence intervals (CIs) for the associations between exposure to air pollutants and ASD stratified by neighborhood income quintile eTable 13. Hazard ratios (HRs) and 95% confidence intervals (CIs) for the associations between exposure to air pollutants and ASD stratified by racialized and newcomer populations quintile

Supplement 2. Data Sharing Statement
