# Supplementary material for: Prenatal Exposure to Fine Particulate Matter Components and Autism Risk in Childhood
Source: JAMA Netw Open. 2025 Oct 23;8(10):e2538882. doi: 10.1001/jamanetworkopen.2025.38882 (PMC12550638; doi:10.1001/jamanetworkopen.2025.38882)
Supplement: Supplement 1. — eMethods. eFigure 1. Flowchart of the inclusion and exclusion of the study population eFigure 2. Directed acyclic graph (DAG) for estimating the effect of ambient air pollution on ASD in children eTable 1. Average prenatal concentrations of ambient PM2.5, its components (BC, dust, NH4+, NO3- , OM, SO4 2-, SS), NO2, and O3 across childhood and pregnancy, and per trimester by ASD status eTable 2. Pearson correlation coefficients between the average prenatal concentrations of PM2.5, its components (BC, dust, NH4+, NO3- , OM, SO4 2-, SS), NO2, and O3 eTable 3. Pearson correlation coefficients between the average first-year-of-life concentrations of PM2.5, its components (BC, dust, NH4+, NO3- , OM, SO4 2-, SS), NO2, and O3 eTable 4. Pearson correlation coefficients between the average prenatal exposure and first year of life concentrations of PM2.5, NO2, and O3 eTable 5. Cumulative hazard ratios (HR) and 95% confidence intervals (CIs) describing the association between prenatal outdoor PM2.5 mass and component concentrations, NO2 and O3 with ASD in single-pollutant models, PM2.5 residual variations-adjusted models, and postnatal exposure-adjusted models over the entire pregnancy and first-year-of-life periods and Distributed Lag Model-identified sensitive windows eTable 6. Association between exposure to average prenatal and first-year-of-life NH4+, NO3- and SO42- and ASD estimated by quantile-based g computation eTable 7. Cumulative hazard ratios (HR) and 95% confidence intervals (CIs) describing the association between prenatal outdoor PM2.5 mass and component concentrations, NO2 and O3 with ASD in single-pollutant modelsa over the entire pregnancy using mixed effect models with two levels of spatial clusters as random effects: census division (equivalent in size to a county) and census tract within census divisions eTable 8. Cumulative hazard ratios (HR) and 95% confidence intervals (CIs) describing the association between prenatal outdoor PM2.5 mass with ASD in singl [file jamanetwopen-e2538882-s001.pdf]

# Supplemental Online Content

Cloutier M, Yu C, Talarico R, et al. Prenatal exposure to fine particulate matter components and autism risk in childhood. *JAMA Netw Open.* 2025;8(10):e2538882. doi:10.1001/jamanetworkopen.2025.38882

**eMethods.**

**eFigure 1.** Flowchart of the Inclusion and Exclusion of the Study Population

**eFigure 2.** Directed Acyclic Graph (DAG) for Estimating the Effect of Ambient Air Pollution on ASD in Children

**eTable 1.** Average prenatal concentrations of ambient $PM_{2.5}$, its components (BC, dust, $NH_4^+$, $NO_3^-$ , OM, $SO_4^{2-}$, SS), $NO_2$, and $O_3$ Across Childhood and Pregnancy, and Per Trimester by ASD Status

**eTable 2.** Pearson Correlation Coefficients Between the Average Prenatal Concentrations of $PM_{2.5}$, Its Components (BC, Dust, $NH_4^+$, $NO_3^-$ , OM, $SO_4^{2-}$, SS), $NO_2$, and $O_3$

**eTable 3.** Pearson Correlation Coefficients Between the Average First-Year-of-Life Concentrations of $PM_{2.5}$, Its Components (BC, Dust, $NH_4^+$, $NO_3^-$ , OM, $SO_4^{2-}$, SS), $NO_2$, and $O_3$

**eTable 4.** Pearson Correlation Coefficients Between the Average Prenatal Exposure and First Year of Life Concentrations of $PM_{2.5}$, $NO_2$, and $O_3$

**eTable 5.** Cumulative Hazard Ratios (HR) and 95% Confidence Intervals (CIs) Describing the Association Between Prenatal Outdoor $PM_{2.5}$ Mass and Component Concentrations, $NO_2$ and $O_3$ With ASD in Single-Pollutant Models, $PM_{2.5}$

© 2025 Cloutier M et al. *JAMA Network Open*.

Residual Variations-Adjusted Models, and Postnatal Exposure-Adjusted Models Over the Entire Pregnancy and First-Year-of-Life Periods and Distributed Lag Model-Identified Sensitive Windows

**eTable 6.** Association Between Exposure to Average Prenatal and First-Year-of-Life $NH_4^+$, $NO_3^-$ and $SO_4^{2-}$ and ASD Estimated by Quantile-Based g Computation

**eTable 7.** Cumulative Hazard Ratios (HR) and 95% Confidence Intervals (CIs) Describing the Association Between Prenatal Outdoor $PM_{2.5}$ Mass and Component Concentrations, $NO_2$ and $O_3$ With ASD in Single-Pollutant Models Over the Entire Pregnancy Using Mixed Effect Models With Two Levels of Spatial Clusters as Random Effects: Census Division (Equivalent in Size to a County) and Census Tract Within Census Divisions

**eTable 8.** Cumulative Hazard Ratios (HR) and 95% Confidence Intervals (CIs) Describing the Association Between Prenatal Outdoor $PM_{2.5}$ Mass With ASD in Single-Pollutant Models Over the Entire Pregnancy Using Different Specifications of Degrees of Freedom in the Distributed Lag Models Over Gestational Weeks

**eTable 9.** Hazard Ratios (HRs) and 95% Confidence Intervals (CIs) for the Associations Between Exposure to Air Pollutants and ASD Stratified by Rural/Urban Residence at Birth

**eTable 10.** Hazard Ratios (HRs) and 95% Confidence Intervals (CIs) for the Associations Between Exposure to Air Pollutants and ASD Stratified by Infant Sex

**eTable 11.** Hazard Ratios (HRs) and 95% Confidence Intervals (CIs) for the Associations Between Exposure to Air Pollutants and ASD Stratified by Maternal Pre-existing Asthma Status

**eTable 12.** Hazard Ratios (HRs) and 95% Confidence Intervals (CIs) for the Associations Between Exposure to Air Pollutants and ASD Stratified by Neighborhood Income Quintile

**eTable 13.** Hazard Ratios (HRs) and 95% Confidence Intervals (CIs) for the Associations Between Exposure to Air Pollutants and ASD Stratified by Racialized and Newcomer Populations Quintile

This supplemental material has been provided by the authors to give readers additional information about their work.

**eMethods**

## 1. Area-Level Socioeconomic Status: Ontario Marginalization Index (ON-Marg)

Area-level socioeconomic status was assessed using the Ontario Marginalization Index (ON-Marg), a census-derived measure capturing multiple aspects of social disadvantages. The index was developed using principal component analysis of selected census indicators and yields four dimensions:

1) **Households and Dwellings:** family and neighborhood stability, housing type, and household composition.
2) **Material Resources:** economic disadvantage, including education, income, and employment.
3) **Age and Labour Force:** concentrations of older adults, children, and individuals outside the labor force, reflecting dependence or disability.
4) **Racialized and Newcomer Populations:** proportion of racialized and recent immigrant populations, reflecting structural and social marginalization.

Each dimension is represented as a standardized factor score (higher values indicate greater marginalization). Quintiles were created for interpretability. Maternal residential postal codes at birth were linked to ON-Marg scores at the dissemination area (DA) level. When DA-level data were missing, values from larger geographic units (census tracts or subdivisions) were used. ON-Marg is available for the 2001, 2006, 2011, 2016, and 2021 censuses and has been widely used in population-level research on social determinants of health. For detailed methodology, indicators, and guidance on using ON-Marg, readers are invited to consult the official user guide: User Guide: 2021 Ontario Marginalization Index

© 2025 Cloutier M et al. *JAMA Network Open*.

## 2. $PM_{2.5}$ Mass and Component Estimates

Prenatal $PM_{2.5}$ Mass and Component concentrations were estimated at a 1 x 1 km spatial resolution (V5.NA.04.02). Estimates were derived by integrating satellite-based aerosol optical depth satellite aerosol optical depth (AOD) retrievals from multiple instruments and algorithms (MISR, MODIS Dark Target, MODIS/SeaWiFS Deep Blue, MODIS MAIAC, VIIRS Dark Target, and VIIRS Deep Blue), a chemical transport model (GEOS-Chem), and ground-based $PM_{2.5}$ mass and composition measurements from the U.S. EPA Air Quality System and the Canadian National Air Pollution Surveillance (NAPS) network using geographically weighted regression.

© 2025 Cloutier M et al. *JAMA Network Open*.

**eFigure 1.** Flowchart of the inclusion and exclusion of the study population**.**

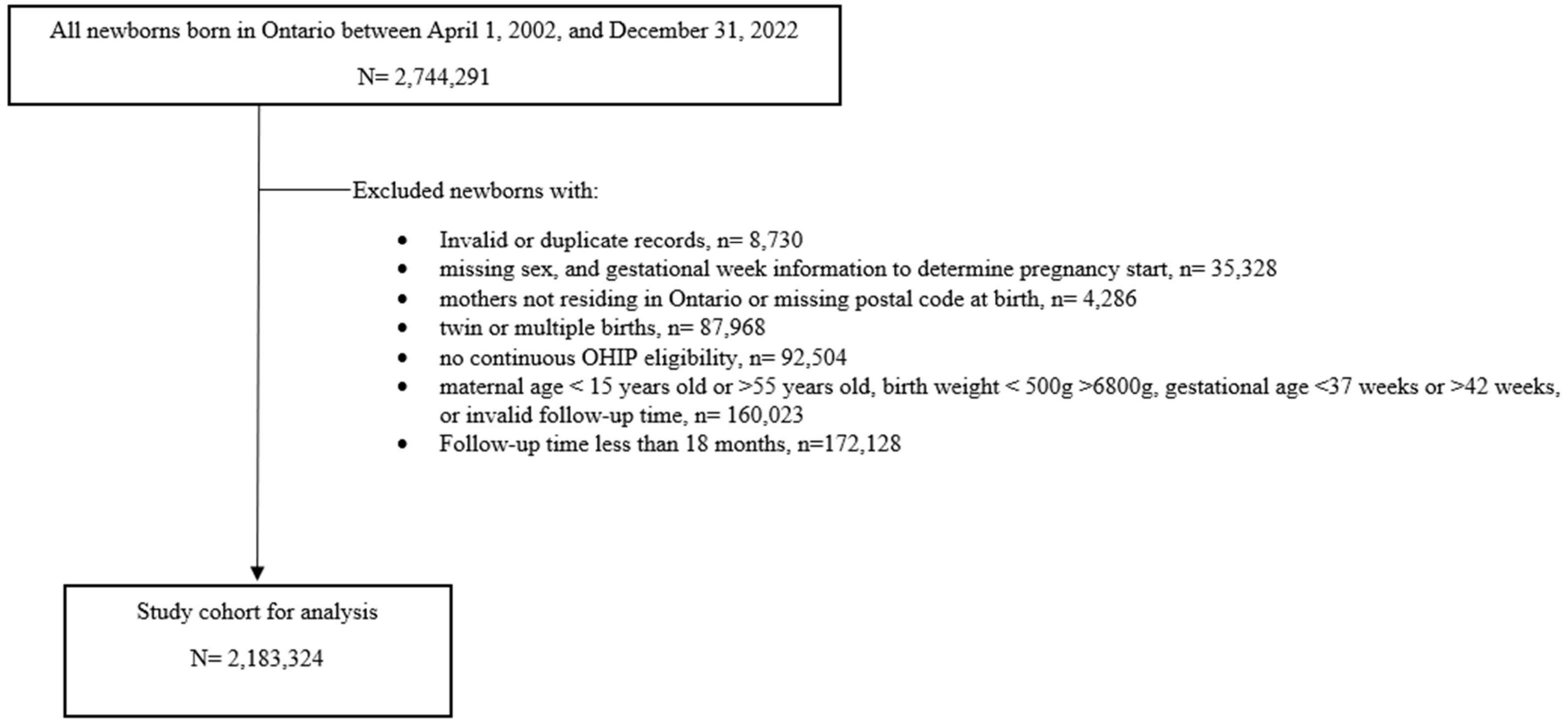


© 2025 Cloutier M et al. *JAMA Network Open*.

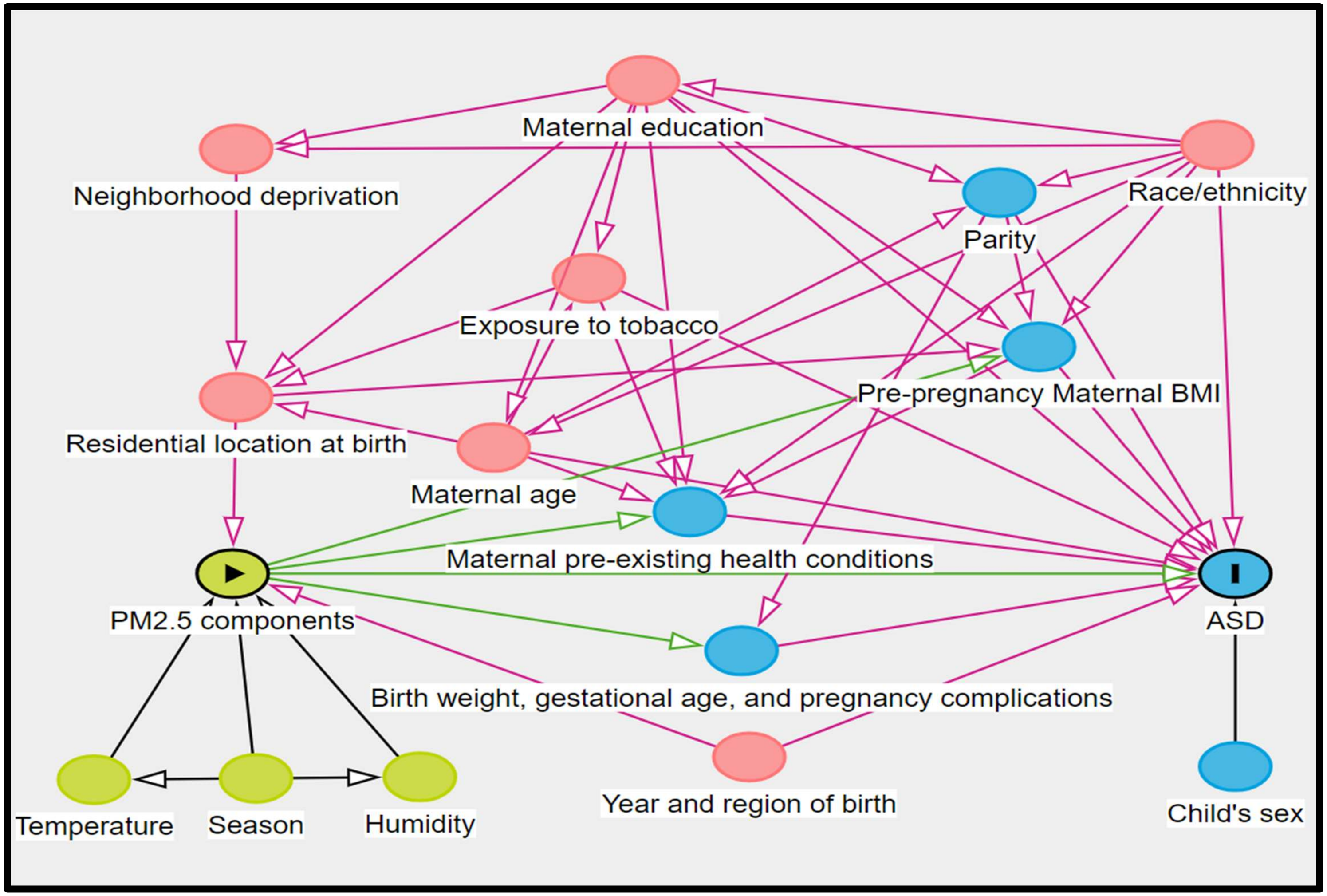


© 2025 Cloutier M et al. *JAMA Network Open*.

**eFigure 2.** Directed acyclic graph (DAG) representing possible confounding of the association between fine particulate matter ($PM_{2.5}$) components (i.e. BC, dust, $NH_4^+$, $NO_3^-$, OM, $SO_4^{2-}$, SS) and autism spectrum disorder (ASD) in children. (1) Nodes: parameters in red are potential confounding factors, parameters in green are causes of the exposure, but not of the outcome, and parameters in blue are causes of the outcome, but not of the exposure. (2) Arrows: causal relationships. Note: BMI: body mass index.

**eTable 1.** Average prenatal concentrations of ambient $PM_{2.5}$, its components (BC, DUST, $NH_4$, $NO_3$, OM, $SO_4$, SS), $NO_2$, and $O_3$ across childhood and pregnancy, and per trimester by ASD status.

| Pollutant | Total<br>Mean ± SD | ASD cases<br>Mean ± SD |
|---|---|---|
| **$PM_{2.5}$ (ug/m$^3$)** | | |
| Pregnancy | 7.82 ± 2.02 | 7.47 ± 1.69 |
| 1st trimester | 7.86 ± 2.48 | 7.48 ± 2.06 |
| 2nd trimester | 7.79 ± 2.35 | 7.46 ± 1.99 |
| 3rd trimester | 7.82 ± 2.53 | 7.45 ± 2.15 |
| Childhood | 7.72 ± 1.92 | 7.24 ± 1.58 |
| **BC (ug/m$^3$)** | | |
| Pregnancy | 0.48 ± 0.19 | 0.48 ± 0.17 |
| 1st trimester | 0.48 ± 0.23 | 0.48 ± 0.21 |
| 2nd trimester | 0.47 ± 0.22 | 0.48 ± 0.20 |
| 3rd trimester | 0.48 ± 0.23 | 0.48 ± 0.21 |
| Childhood | 0.47 ± 0.18 | 0.46 ± 0.15 |
| **Dust (ug/m$^3$)** | | |
| Pregnancy | 0.48 ± 0.18 | 0.49 ± 0.17 |
| 1st trimester | 0.47 ± 0.21 | 0.49 ± 0.20 |
| 2nd trimester | 0.47 ± 0.21 | 0.49 ± 0.19 |
| 3rd trimester | 0.48 ± 0.22 | 0.49 ± 0.21 |
| Childhood | 0.47 ± 0.18 | 0.48 ± 0.16 |
| **$NH_4^+$ (ug/m$^3$)** | | |
| Pregnancy | 0.74 ± 0.29 | 0.69 ± 0.25 |
| 1st trimester | 0.75 ± 0.33 | 0.70 ± 0.30 |
| 2nd trimester | 0.74 ± 0.32 | 0.69 ± 0.29 |
| 3rd trimester | 0.73 ± 0.34 | 0.68 ± 0.30 |
| Childhood | 0.73 ± 0.28 | 0.66 ± 0.23 |
| **$NO_3^-$ (ug/m$^3$)** | | |

© 2025 Cloutier M et al. *JAMA Network Open*.

| | | |
|---|---|---|
| Pregnancy | 0.96 ± 0.44 | 0.93 ± 0.40 |
| 1st trimester | 0.97 ± 0.73 | 0.96 ± 0.74 |
| 2nd trimester | 0.97 ± 0.71 | 0.93 ± 0.70 |
| 3rd trimester | 0.93 ± 0.75 | 0.89 ± 0.73 |
| Childhood | 0.93 ± 0.35 | 0.90 ± 0.29 |
| **OM ($ug/m^3$)** | | |
| Pregnancy | 4.14 ± 1.07 | 4.08 ± 0.91 |
| 1st trimester | 4.14 ± 1.53 | 4.04 ± 1.32 |
| 2nd trimester | 4.11 ± 1.44 | 4.07 ± 1.28 |
| 3rd trimester | 4.18 ± 1.56 | 4.12 ± 1.36 |
| Childhood | 4.12 ± 0.96 | 4.00 ± 0.79 |
| **$SO_4^{2-}$ ($ug/m^3$)** | | |
| Pregnancy | 1.71 ± 0.58 | 1.62 ± 0.49 |
| 1st trimester | 1.72 ± 0.72 | 1.61 ± 0.61 |
| 2nd trimester | 1.70 ± 0.68 | 1.62 ± 0.59 |
| 3rd trimester | 1.72 ± 0.73 | 1.62 ± 0.65 |
| Childhood | 1.70 ± 0.55 | 1.55 ± 0.45 |
| **SS ($ug/m^3$)** | | |
| Pregnancy | 0.09 ± 0.04 | 0.09 ± 0.04 |
| 1st trimester | 0.09 ± 0.06 | 0.09 ± 0.06 |
| 2nd trimester | 0.09 ± 0.06 | 0.09 ± 0.06 |
| 3rd trimester | 0.09 ± 0.06 | 0.09 ± 0.06 |
| Childhood | 0.09 ± 0.04 | 0.09 ± 0.04 |
| **$NO_2$ (ppb)** | | |
| Pregnancy | 5.68 ± 2.97 | 5.82 ± 3.18 |
| 1st trimester | 5.67 ± 3.43 | 5.90 ± 3.70 |
| 2nd trimester | 5.71 ± 3.39 | 5.83 ± 3.62 |
| 3rd trimester | 5.64 ± 3.45 | 5.73 ± 3.64 |
| Childhood | 5.68 ± 2.83 | 5.82 ± 3.01 |
| **$O_3$ (ppb)** | | |
| Pregnancy | 46.29 ± 9.56 | 45.33 ± 9.43 |
| 1st trimester | 45.84 ± 11.74 | 45.07 ± 11.63 |
| 2nd trimester | 46.52 ± 11.80 | 45.56 ± 11.58 |

© 2025 Cloutier M et al. *JAMA Network Open*.

| | | |
|---|---|---|
| 3rd trimester | 46.74 ± 12.16 | 45.29 ± 11.91 |
| Childhood | 46.22 ± 9.15 | 45.00 ± 9.12 |

Abbreviations: $PM_{2.5}$, fine particulate matter with a diameter ≤ 2.5 µm; BC, black carbon; $NH_4^+$, ammonium; $NO_3^-$ , nitrate; OM, organic matter; $SO_4^{2-}$, sulphate; SS, sea salt; $NO_2$, nitrogen dioxide; $O_3$, ozone; ASD, autism spectrum disorder.

**eTable 2.** Pearson correlation coefficients between the average prenatal concentrations of $PM_{2.5}$, its components (BC, dust, $NH_4^+$, $NO_3^-$, OM, $SO_4^{2-}$, SS), $NO_2$, and $O_3$.

| **Air pollutants** | **$PM_{2.5}$** | **BC** | **DUST** | **$NH_4$** | **$NO_3$** | **OM** | **$SO_4$** | **SS** | **$NO_2$** | **$O_3$** |
|---|---|---|---|---|---|---|---|---|---|---|
| **$PM_{2.5}$** | 1.00 | | | | | | | | | |
| **BC** | 0.75 | 1.00 | | | | | | | | |
| **DUST** | 0.65 | 0.73 | 1.00 | | | | | | | |
| **$NH_4^+$** | 0.81 | 0.54 | 0.41 | 1.00 | | | | | | |
| **$NO_3^-$** | 0.54 | 0.27 | 0.35 | 0.78 | 1.00 | | | | | |
| **OM** | 0.66 | 0.83 | 0.64 | 0.28 | 0.01 | 1.00 | | | | |
| **$SO_4^{2-}$** | 0.78 | 0.64 | 0.45 | 0.78 | 0.29 | 0.46 | 1.00 | | | |
| **SS** | 0.61 | 0.48 | 0.56 | 0.65 | 0.74 | 0.28 | 0.37 | 1.00 | | |
| **$NO_2$** | 0.17 | 0.19 | 0.16 | 0.19 | 0.27 | 0.11 | 0.07 | 0.22 | 1.00 | |
| **$O_3$** | -0.11 | -0.23 | -0.23 | 0.04 | -0.01 | -0.29 | 0.07 | -0.09 | -0.09 | 1.00 |

Abbreviations: $PM_{2.5}$, fine particulate matter with a diameter ≤ 2.5 µm; BC, black carbon; $NH_4^+$, ammonium; $NO_3^-$, nitrate; OM, organic matter; $SO_4^{2-}$, sulphate; SS, sea salt; $NO_2$, nitrogen dioxide; $O_3$, ozone.

© 2025 Cloutier M et al. *JAMA Network Open*.

**eTable 3.** Pearson correlation coefficients between the average first-year-of-life concentrations of $PM_{2.5}$, its components (BC, dust, $NH_4^+$, $NO_3^-$, OM, $SO_4^{2-}$, SS), $NO_2$, and $O_3$.

| **Air pollutants** | **$PM_{2.5}$** | **BC** | **DUST** | **$NH_4$** | **$NO_3$** | | **OM** | **$SO_4$** | **SS** | **$NO_2$** | **$O_3$** |
|---|---|---|---|---|---|---|---|---|---|---|---|
| **$PM_{2.5}$** | 1.00 | | | | | | | | | | |
| **BC** | 0.77 | 1.00 | | | | | | | | | |
| **DUST** | 0.66 | 0.74 | 1.00 | | | | | | | | |
| **$NH_4^+$** | 0.84 | 0.65 | 0.46 | 1.00 | | | | | | | |
| **$NO_3^-$** | 0.78 | 0.62 | 0.62 | 0.84 | 1.00 | | | | | | |
| **OM** | 0.70 | 0.83 | 0.66 | 0.41 | 0.41 | | 1.00 | | | | |
| **$SO_4^{2-}$** | 0.79 | 0.61 | 0.43 | 0.93 | 0.69 | | 0.36 | 1.00 | | | |
| **SS** | 0.69 | 0.65 | 0.67 | 0.63 | 0.76 | | 0.51 | 0.53 | 1.00 | | |
| **$NO_2$** | 0.22 | 0.29 | 0.22 | 0.16 | 0.19 | | 0.26 | 0.15 | 0.18 | 1.00 | |
| **$O_3$** | -0.11 | -0.23 | -0.26 | 0.03 | -0.04 | | -0.30 | 0.09 | -0.13 | -0.14 | 1.00 |

Abbreviations: $PM_{2.5}$, fine particulate matter with a diameter ≤ 2.5 µm; BC, black carbon; $NH_4^+$, ammonium; $NO_3^-$, nitrate; OM, organic matter; $SO_4^{2-}$, sulphate; SS, sea salt; $NO_2$, nitrogen dioxide; $O_3$, ozone.

© 2025 Cloutier M et al. *JAMA Network Open*.

**eTable 4.** Pearson correlation coefficients between the average prenatal exposure and first-year-of-life concentrations of $PM_{2.5}$, $NO_2$, and $O_3$.

| Air pollutants | Prenatal $PM_{2.5}$ | 1st year $PM_{2.5}$ | Prenatal $NO_2$ | 1st year $NO_2$ | Prenatal $O_3$ | 1st year $O_3$ |
|---|---|---|---|---|---|---|
| **Prenatal $PM_{2.5}$** | 1.00 | | | | | |
| **1st year $PM_{2.5}$** | 0.84 | 1.00 | | | | |
| **Prenatal $NO_2$** | 0.13 | 0.16 | 1.00 | | | |
| **1st year $NO_2$** | 0.16 | 0.18 | 0.75 | 1.00 | | |
| **Prenatal $O_3$** | 0.03 | 0.03 | -0.07 | -0.11 | 1.00 | |
| **1st year $O_3$** | 0.07 | 0.05 | -0.09 | -0.11 | 0.80 | 1.00 |

Abbreviations: $PM_{2.5}$, fine particulate matter with a diameter ≤ 2.5 µm; $NO_2$,, nitrogen dioxide; $O_3$, ozone.

© 2025 Cloutier M et al. *JAMA Network Open*.

**eTable 5**. Cumulative hazard ratios (HR) and 95% confidence intervals describing the association between prenatal outdoor $PM_{2.5}$ mass and component concentrations, $NO_2$ and $O_3$ with ASD in single-pollutant models[a], $PM_{2.5}$ residual variations-adjusted models[b], and early life exposure-adjusted models[c] over the entire pregnancy and first year life periods and Distributed Lag Model-identified sensitive windows.

| Pollutant | Adjusted model | Prenatal period | First year of life |
|---|---|---|---|
| $PM_{2.5}$ | Single-pollutant[a] | 1.055 (0.990 – 1.123) | 0.873 (0.835 – 0.912) |
| | + Mutually adjusted[c] | 1.148 (1.073 – 1.229)[d] | 0.873 (0.835 – 0.912) |
| $SO_4^{2-}$ | Single-pollutant[a] | 1.100 (1.021 – 1.184) | 0.914 (0.866 – 0.965) |
| | + $PM_{2.5}$[b] | 1.158 (1.082 – 1.240) | 1.137 (1.038 – 1.245) |
| | + Mutually adjusted[c] | 1.154 (1.062 – 1.254)[e] | 0.914 (0.827 – 1.013) |
| $NH_4^+$ | Single-pollutant[a] | 1.030 (0.939 – 1.130) | 0.919 (0.873 – 0.966) |
| | + $PM_{2.5}$[b] | 1.133 (1.045 – 1.228) | 1.170 (1.067 – 1.284) |
| | + Mutually adjusted[c] | 1.115 (1.009 – 1.232)[f] | 0.929 (0.831 – 1.066) |
| $NO_3^-$ | Single-pollutant[a] | 1.057 (0.960 – 1.163) | 0.989 (0.955 – 1.025) |
| | + $PM_{2.5}$[b] | 1.133 (1.041 – 1.233) | 1.200 (1.134 – 1.269) |
| | + Mutually adjusted[c] | 1.076 (0.963 – 1.202) | 0.957 (0.844 – 1.125) |
| SS | Single-pollutant[a] | 1.053 (0.996 – 1.114) | 1.007 (0.974 – 1.042) |
| | + $PM_{2.5}$[b] | 1.044 (0.985 – 1.108) | 1.098 (1.055 – 1.144) |
| | + Mutually adjusted[c] | 1.045 (0.962 – 1.134) | 1.000 (0.921 – 1.090) |
| BC | Single-pollutant[a] | 0.862 (0.813 – 0.913) | 0.864 (0.826 – 0.904) |
| | + $PM_{2.5}$[b] | 0.914 (0.862 – 0.969) | 0.909 (0.859 – 0.961) |
| | + Mutually adjusted[c] | 0.983 (0.915 – 1.058) | 0.944 (0.870 – 1.022) |
| dust | Single-pollutant[a] | 1.012 (0.965 – 1.061) | 0.984 (0.944 – 1.026) |
| | + $PM_{2.5}$[b] | 0.947 (0.898 – 0.999) | 1.094 (1.039 – 1.151) |
| | + Mutually adjusted[c] | 1.003 (0.935 – 1.076) | 0.939 (0.875 – 1.007) |
| OM | Single-pollutant[a] | 0.930 (0.859 – 1.005) | 0.877 (0.838 – 0.917) |
| | + $PM_{2.5}$[b] | 0.872 (0.804 – 0.946) | 0.932 (0.879 – 0.989) |
| | + Mutually adjusted[c] | 0.931 (0.838 – 1.033) | 0.969 (0.870 – 1.078) |
| $NO_2$ | Single-pollutant[a] | 1.011 (0.983 – 1.039) | 1.015 (0.991 – 1.040) |

© 2025 Cloutier M et al. *JAMA Network Open*.

| | | | |
|---|---|---|---|
| | + $PM_{2.5}$[b] | 1.011 (0.982 – 1.040) | 1.016 (0.991 – 1.041) |
| | + Mutually adjusted[c] | 0.989 (0.941 – 1.039) | 0.997 (0.948 – 1.048) |
| $O_3$ | Single-pollutant[a] | 1.092 (1.048 – 1.137) | 1.055 (1.030 – 1.081) |
| | + $PM_{2.5}$[b] | 1.100 (1.055 – 1.146) | 1.059 (1.034 – 1.085) |
| | + Early life adjusted[c] | 1.057 (0.978 – 1.141)[g] | 1.090 (1.013 – 1.172) |

Note: All hazard ratios are scaled by the interquartile range of each pollutant: $PM_{2.5}$ (3.50 µg/m$^3$), $SO_4^{2-}$ (0.95 ug/m$^3$), $NH_4^+$ (0.60 ug/m$^3$), $NO_3^-$ (1.10 ug/m$^3$), SS (0.10 ug/m$^3$), BC (0.30 ug/m$^3$), dust (0.30 ug/m$^3$), OM (2.20 ug/m$^3$), $NO_2$ (4.91 ppb), $O_3$ (19.13 ppb).

[a] All models were adjusted for infant sex, birth weight, maternal parity, maternal pre-pregnancy history of hypertension and diabetes (≥ 1 diagnosis), season of birth (spring, summer, fall, winter), maternal age, area-level socioeconomic status variables, urbanicity (urban vs rural), geographic indicators, birth year, and calendar week in a year of birth. Abbreviations: ASD, autism spectrum disorder; $PM_{2.5}$, fine particulate matter with a diameter ≤ 2.5 µm; BC, black carbon; $NH_4^+$, ammonium; $NO_3^-$, nitrate; OM, organic matter; $SO_4^{2-}$, sulphate; SS, sea salt; $NO_2$, nitrogen dioxide; $O_3$, ozone.
[b] Includes all variables in the single-pollutant model plus the $PM_{2.5}$ residuals (i.e., $PM_{2.5}$ ~ each component) for models for single components or $PM_{2.5}$ total mass for models for $NO_2$ and $O_3$.
[c] Includes all variables from the previous model (i.e., $PM_{2.5}$ adjusted) plus exposure to $PM_{2.5}$ and the selected pollutant during both the prenatal and first year of life periods.
[d] HR for the Distributed Lag Model-identified sensitive windows for $PM_{2.5}$ from 14 – 32 weeks of gestation. 1.122 (1.070 – 1.177)
[e] HR for the Distributed Lag Model-identified sensitive windows for $SO_4^{2-}$ from 23 – 36 weeks of gestation. 1.107 (1.042 – 1.177)
[f] HR for the Distributed Lag Model-identified sensitive windows for $NH_4^+$ from 21 – 34 weeks of gestation 1.110 (1.039 – 1.186)
[g] HR for the Distributed Lag Model-identified sensitive windows for $O_3$ from 26 – 30 weeks of gestation. 1.028 (1.002 – 1.054)

© 2025 Cloutier M et al. *JAMA Network Open*.

**eTable 6.** Association[a] between exposure to average prenatal and first-year-of-life $NH_4^+$, $NO_3^-$ and $SO_4^{2-}$ and ASD estimated by quantile-based g computation.

| **Association** | Contribution to association, % | Overall HR (95% CI)[a] |
|---|---|---|
| Positive association with ASD | | |
| 1st year $NH_4^+$ | 38.3 | |
| Prenatal $NO_3^-$ | 7.2 | |
| Prenatal $SO_4^2$ | 2.6 | |
| 1st year $SO_4^{2-}$ | 51.9 | 1.022 (0.963-1.084) |
| Negative association with ASD | | |
| Prenatal $NH_4^+$ | 42.9 | |
| 1st year $NO_3^-$ | 57.1 | |

Note: All models were adjusted for infant sex, birth weight, maternal parity, maternal pre-pregnancy history of hypertension and diabetes (≥ 1 diagnosis), season of birth (spring, summer, fall, winter), maternal age, area-level socioeconomic status variables, geographic indicators, birth year, calendar week in a year of birth, $PM_{2.5}$ residuals (i.e., $PM_{2.5} \sim$ each component) for models for single components. Abbreviations: ASD, autism spectrum disorder; $NH_4^+$, ammonium; $NO_3^-$, nitrate; $SO_4^{2-}$, sulphate.

[a]The overall association between exposure to $PM_{2.5}$ and ASD per quartile increase in $PM_{2.5}$.

© 2025 Cloutier M et al. *JAMA Network Open*.

**eTable 7**. Cumulative hazard ratios[a] (HR) and 95% confidence intervals (CIs) describing the association between prenatal outdoor $PM_{2.5}$ mass and component concentrations, $NO_2$ and $O_3$ with ASD in single-pollutant models[a] over the entire pregnancy using mixed effect models with two levels of spatial clusters as random effects: census division (equivalent in size to a county) and census tract within census divisions.

| **Pollutant** | **Prenatal period** |
|---|---|
| $PM_{2.5}$ | 1.051 (0.988 – 1.121) |
| $SO_4^{2-}$ | 1.098 (1.019 – 1.182) |
| $NH_4^+$ | 1.030 (0.939 – 1.130) |
| $NO_3^-$ | 1.050 (0.954 – 1.158) |
| SS | 1.053 (0.996 – 1.114) |
| BC | 0.873 (0.825 – 0.922) |
| dust | 1.011 (0.964 – 1.059) |
| OM | 0.941 (0.870 – 1.016) |
| $NO_2$ | 1.009 (0.976 – 1.027) |
| $O_3$ | 1.091 (1.046 – 1.135) |

Note: All hazard ratios are scaled by the interquartile range of each pollutant: $PM_{2.5}$ (3.50 µg/m$^3$), $SO_4^{2-}$ (0.95 ug/m$^3$), $NH_4^+$ (0.60 ug/m$^3$), $NO_3^-$ (1.10 ug/m$^3$), SS (0.10 ug/m$^3$), BC (0.30 ug/m$^3$), dust (0.30 ug/m$^3$), OM (2.20 ug/m$^3$), $NO_2$ (4.91 ppb), $O_3$ (19.13 ppb).

[a] All models were adjusted for infant sex, birth weight, maternal parity, maternal pre-pregnancy history of hypertension and diabetes (≥ 1 diagnosis), season of birth (spring, summer, fall, winter), maternal age, area-level socioeconomic status variables, urbanicity (urban vs rural), geographic indicators, birth year, and calendar week in a year of birth. Abbreviations: ASD, autism spectrum disorder; $PM_{2.5}$, fine particulate matter with a diameter ≤ 2.5 µm; BC, black carbon; $NH_4^+$, ammonium; $NO_3^-$, nitrate; OM, organic matter; $SO_4^{2-}$, sulphate; SS, sea salt; $NO_2$, nitrogen dioxide; $O_3$, ozone.

© 2025 Cloutier M et al. *JAMA Network Open*.

**eTable 8**. Cumulative hazard ratios[a] (HR) and 95% confidence intervals (CIs) describing the association between prenatal outdoor $PM_{2.5}$ mass with ASD in single-pollutant models[a] over the entire pregnancy using different specifications of degrees of freedom in the distributed lag models over gestational weeks.

| **Degrees of freedom** | **Prenatal period** |
|---|---|
| 3df | 1.052 (0.990 – 1.122) |
| 4df | 1.053 (0.990 – 1.123) |
| 5df | 1.051 (0.988 – 1.121) |

Note: All hazard ratios are scaled by the interquartile range of the pollutant: $PM_{2.5}$ (3.50 µg/$m^3$).

[a] All models were adjusted for infant sex, birth weight, maternal parity, maternal pre-pregnancy history of hypertension and diabetes (≥ 1 diagnosis), season of birth (spring, summer, fall, winter), maternal age, area-level socioeconomic status variables, urbanicity (urban vs rural), geographic indicators, birth year, and calendar week in a year of birth. Abbreviations: ASD, autism spectrum disorder; $PM_{2.5}$, fine particulate matter with a diameter ≤ 2.5 µm; BC, black carbon; $NH_4^+$, ammonium; $NO_3^-$, nitrate; OM, organic matter; $SO_4^{2-}$, sulphate; SS, sea salt; $NO_2$, nitrogen dioxide; $O_3$, ozone.

© 2025 Cloutier M et al. *JAMA Network Open*.

**eTable 9.** Hazard ratios (HRs) and 95% confidence intervals (CIs) for the associations between prenatal exposure to air pollutants and ASD stratified by rural/urban residence at birth.

| Exposure | Rural | | Urban | | p-value[a] |
|---|---|---|---|---|---|
| | Cohort size (cases) | HR (95% CI) | Cohort size (cases) | HR (95% CI) | |
| $PM_{2.5}$ | 260,115 (1,273) | 1.104 (0.939 – 1.299) | 1,923,164 (17,965) | 1.093 (1.049 – 1.139) | 0.0006 |
| $SO_4^{2-}$ | | 1.102 (0.921 – 1.317) | | 1.152 (1.099 – 1.208) | 0.0271 |
| $NH_4^+$ | | 1.269 (0.984 – 1.639) | | 1.155 (1.087 – 1.227) | 0.0160 |
| $NO_2$ | | 0.866 (0.593 – 1.265) | | 1.029 (0.989 – 1.072) | 0.0219 |
| $O_3$ | | 1.117 (0.959 – 1.299) | | 1.035 (0.984 – 1.088) | 0.3873 |

Note: All models were adjusted for infant sex, birth weight, maternal parity, maternal pre-pregnancy history of hypertension and diabetes (≥ 1 diagnosis), season of birth (spring, summer, fall, winter), maternal age, area-level socioeconomic status variables, geographic indicators, birth year, calendar week in a year of birth, $PM_{2.5}$ residuals (i.e., $PM_{2.5}$ ~ each component) for models for single components or $PM_{2.5}$ total mass for models for $NO_2$ and $O_3$ and exposures during the first year of life. Abbreviations: ASD, autism spectrum disorder; $PM_{2.5}$, fine particulate matter with a diameter ≤ 2.5 µm; $NH_4^+$, ammonium; $SO_4^{2-}$, sulphate; $NO_2$, nitrogen dioxide; $O_3$, ozone.

[a] P-values correspond to the multiplicative interaction term between the exposure and effect modifier (from a separate model).

© 2025 Cloutier M et al. *JAMA Network Open*.

**eTable 10.** Hazard ratios (HRs) and 95% confidence intervals (CIs) for the associations between prenatal exposure to air pollutants and ASD stratified by infant sex.

| Exposure | Male | | Female | | p-value[a] |
|---|---|---|---|---|---|
| | Cohort size (cases) | HR (95% CI) | Cohort size (cases) | HR (95% CI) | |
| $PM_{2.5}$ | 1,115,324 (14,937) | 1.099 (1.052 – 1.149) | 1,067,955 (4,301) | 1.057 (0.971 – 1.149) | 1.104E-09 |
| $SO_4^{2-}$ | | 1.155 (1.098 – 1.216) | | 1.116 (1.009 – 1.235) | 2.514E-08 |
| $NH_4^+$ | | 1.173 (1.098 – 1.254) | | 1.086 (0.955 – 1.234) | 1.039E-08 |
| $NO_2$ | | 1.027 (0.982 – 1.074) | | 1.018 (0.936 – 1.108) | 0.773 |
| $O_3$ | | 1.037 (0.982 – 1.094) | | 1.061 (0.959 – 1.173) | 0.709 |

Note: All models were adjusted for infant sex, birth weight, maternal parity, maternal pre-pregnancy history of hypertension and diabetes (≥ 1 diagnosis), season of birth (spring, summer, fall, winter), maternal age, area-level socioeconomic status variables, geographic indicators, birth year, calendar week in a year of birth, $PM_{2.5}$ residuals (i.e., $PM_{2.5}$ ~ each component) for models for single components or $PM_{2.5}$ total mass for models for $NO_2$ and $O_3$ and exposures during the first year of life. Abbreviations: ASD, autism spectrum disorder; $PM_{2.5}$, fine particulate matter with a diameter ≤ 2.5 µm; $NH_4^+$, ammonium; $SO_4^{2-}$, sulphate; $NO_2$, nitrogen dioxide; $O_3$, ozone.

[a] P-values correspond to the multiplicative interaction term between the exposure and effect modifier (from a separate model).

© 2025 Cloutier M et al. *JAMA Network Open*.

**eTable 11.** Hazard ratios (HRs) and 95% confidence intervals (CIs) for the associations between prenatal exposure to air pollutants and ASD stratified by maternal pre-existing asthma status.

| Exposure | Asthma | | No asthma | | p-value[a] |
|---|---|---|---|---|---|
| | Cohort size (cases) | HR (95% CI) | Cohort size (cases) | HR (95% CI) | |
| $PM_{2.5}$ | 310,152 (3,099) | 1.119 (1.013 – 1.237) | 1,873,127 (16,139) | 1.083 (1.038 – 1.131) | 0.571 |
| $SO_4^{2-}$ | | 1.155 (1.028 – 1.298) | | 1.142 (1.087 – 1.200) | 0.926 |
| $NH_4^+$ | | 1.186 (1.021 – 1.378) | | 1.146 (1.075 – 1.221) | 0.179 |
| $NO_2$ | | 1.091 (0.980 – 1.214) | | 1.014 (0.971 – 1.058) | 0.968 |
| $O_3$ | | 1.064 (0.947 – 1.196) | | 1.037 (0.984 – 1.092) | 0.019 |

Note: All models were adjusted for infant sex, birth weight, maternal parity, maternal pre-pregnancy history of hypertension and diabetes (≥ 1 diagnosis), season of birth (spring, summer, fall, winter), maternal age, area-level socioeconomic status variables, geographic indicators, birth year, calendar week in a year of birth, $PM_{2.5}$ residuals (i.e., $PM_{2.5}$ ~ each component) for models for single components or $PM_{2.5}$ total mass for models for $NO_2$ and $O_3$ and exposures during the first year of life. Abbreviations: ASD, autism spectrum disorder; $PM_{2.5}$, fine particulate matter with a diameter ≤ 2.5 µm; $NH_4^+$, ammonium; $SO_4^{2-}$, sulphate; $NO_2$, nitrogen dioxide; $O_3$, ozone.

[a] P-values correspond to the multiplicative interaction term between the exposure and effect modifier (from a separate model).

© 2025 Cloutier M et al. *JAMA Network Open*.

**eTable 12.** Hazard ratios (HRs) and 95% confidence intervals (CIs) for the associations between prenatal exposure to air pollutants and ASD stratified by neighborhood income quintile.

| Exposure | Quintile 1 | | Quintile 2 | | Quintile 3 | | Quintile 4 | | Quintile 5 |
|---|---|---|---|---|---|---|---|---|---|
| | HR (95% CI) | p-value[a] | HR (95% CI) | p-value[a] | HR (95% CI) | p-value[a] | HR (95% CI) | p-value[a] | HR (95% CI) |
| $PM_{2.5}$ | 1.115 (1.037 – 1.199) | 0.899 | 0.991 (0.909 – 1.079) | 0.002 | 1.175 (1.075 – 1.284) | 0 | 1.129(1.028 – 1.242) | 5.00E-04 | 1.033 (0.922 – 1.158) |
| $SO_4^{2}$ | 1.217 (1.119 – 1.323) | 0.334 | 0.968 (0.874 – 1.072) | 0.008 | 1.198 (1.081 – 1.328) | 0 | 1.263 (1.133 – 1.407) | 1.00E-04 | 1.089 (0.954 – 1.244) |
| $NH_4^+$ | 1.169 (1.051 – 1.299) | 0.664 | 1.027 (0.904 – 1.167) | 0.001 | 1.275 (1.115 – 1.458) | 0 | 1.305 (1.129 – 1.508) | 0 | 1.0312 (0.866 – 1.229) |
| $NO_2$ | 0.997 (0.937 – 1.061) | 0.317 | 1.009 (0.928 – 1.098) | 0.706 | 1.041 (0.939 – 1.155) | 0.721 | 1.030 (0.914 – 1.162) | 0.620 | 1.054 (0.913 – 1.215) |
| $O_3$ | 1.043 (0.944 – 1.152) | 0.499 | 0.949 (0.856 – 1.053) | 0.095 | 1.041 (0.939 – 1.155) | 0.795 | 1.137 (1.021 – 1.266) | 0.070 | 1.117 (0.975 – 1.279) |

Note: All models were adjusted for infant sex, birth weight, maternal parity, maternal pre-pregnancy history of hypertension and diabetes (≥ 1 diagnosis), season of birth (spring, summer, fall, winter), maternal age, area-level socioeconomic status variables, geographic indicators, birth year, calendar week in a year of birth, $PM_{2.5}$ residuals (i.e., $PM_{2.5} \sim$ each component) for models for single components or $PM_{2.5}$ total mass for models for $NO_2$ and $O_3$ and exposures during the first year of life. Abbreviations: ASD, autism spectrum disorder; $PM_{2.5}$, fine particulate matter with a diameter ≤ 2.5 µm; $NH_4^+$, ammonium; $SO_4^{2-}$, sulphate; $NO_2$, nitrogen dioxide; $O_3$, ozone.

[a] P-values correspond to the multiplicative interaction term between the exposure and effect modifier (from a separate model).

© 2025 Cloutier M et al. *JAMA Network Open*.

**eTable 13.** Hazard ratios[a] (HRs) and 95% confidence intervals (CIs) for the associations between prenatal exposure to air pollutants and ASD stratified by racialized and newcomer populations quintile.

| Exposure | Quintile 1 | | Quintile 2 | | Quintile 3 | | Quintile 4 | | Quintile 5 |
|---|---|---|---|---|---|---|---|---|---|
| | HR (95% CI) | p-value[a] | HR (95% CI) | p-value[a] | HR (95% CI) | p-value[a] | HR (95% CI) | p-value[a] | HR (95% CI) |
| $PM_{2.5}$ | 1.088 (0.948 – 1.249) | 0.0096 | 1.022 (0.903 – 1.157) | 0.0553 | 1.157 (1.043 – 1.283) | 0.0417 | 1.209 (1.111 – 1.315) | 0 | 1.059 (0.998 – 1.124) |
| $SO_4{}^{2}$ | 1.044 (0.899 – 1.212) | 0.0611 | 1.048 (0.912 – 1.203) | 0.923 | 1.258 (1.122 – 1.410) | 0.3227 | 1.183(1.072 – 1.304) | 0 | 1.146 (1.065 – 1.233) |
| $NH_4^+$ | 1.175 (0.959 – 1.440) | 0.0282 | 0.966 (0.804 – 1.160) | 0.0091 | 1.240 (1.065 – 1.445) | 0.0334 | 1.243 (1.097 – 1.409) | 0 | 1.162 (1.064 – 1.271) |
| $NO_2$ | 0.995 (0.805 – 1.232) | 0.3768 | 0.982 (0.812 – 1.186) | 0.0854 | 1.104 (0.964 – 1.264) | 0.0239 | 1.055 (0.960 –1.159) | 2.00E-04 | 1.012 (0.962 – 1.066) |
| $O_3$ | 1.042 (0.900 – 1.205) | 0.8909 | 1.018 (0.884 – 1.172) | 0.4344 | 1.029 (0.907 – 1.167) | 0.6094 | 1.089 (0.981 – 1.209) | 0.9428 | 1.026 (0.953 – 1.105) |

Note: All models were adjusted for infant sex, birth weight, maternal parity, maternal pre-pregnancy history of hypertension and diabetes (≥ 1 diagnosis), season of birth (spring, summer, fall, winter), maternal age, area-level socioeconomic status variables, geographic indicators, birth year, calendar week in a year of birth, $PM_{2.5}$ residuals (i.e., $PM_{2.5} \sim$ each component) for models for single components or $PM_{2.5}$ total mass for models for $NO_2$ and $O_3$ and exposures during the first year of life. Abbreviations: ASD, autism spectrum disorder; $PM_{2.5}$, fine particulate matter with a diameter ≤ 2.5 µm; $NH_4^+$, ammonium; $SO_4^{2-}$, sulphate; $NO_2$, nitrogen dioxide; $O_3$, ozone.

[a] P-values correspond to the multiplicative interaction term between the exposure and effect modifier (from a separate model).

© 2025 Cloutier M et al. *JAMA Network Open*.
