# Supplementary material for: Prenatal Exposure to Fine Particulate Matter Components and Autism Risk in Childhood
Source: JAMA Netw Open. 2025 Oct 23;8(10):e2538882. doi: 10.1001/jamanetworkopen.2025.38882 (PMC12550638; doi:10.1001/jamanetworkopen.2025.38882)
Supplement: Supplement 2. — Data Sharing Statement [file jamanetwopen-e2538882-s002.pdf]

# Data Sharing Statement

Cloutier. Prenatal Exposure to Fine Particulate Matter Components and Autism Risk in Childhood. *JAMA Netw Open*. Published October 23, 2025.
doi:10.1001/jamanetworkopen.2025.38882

**Data**
**Data available:** No

**Additional Information**
**Explanation for why data not available:** The individual level data is not available for sharing due to confidentiality agreements.
